# Two Fixed Ratio Dilutions for Soil Salinity Monitoring in Hypersaline Wetlands

**DOI:** 10.1371/journal.pone.0126493

**Published:** 2015-05-22

**Authors:** Juan Herrero, David C. Weindorf, Carmen Castañeda

**Affiliations:** 1 Estación Experimental de Aula Dei, CSIC, Ave. Montañana 1005, 50059 Zaragoza, Spain; 2 Department of Plant and Soil Science, Texas Tech University, Lubbock, Texas 79409, United States of America; DOE Pacific Northwest National Laboratory, UNITED STATES

## Abstract

Highly soluble salts are undesirable in agriculture because they reduce yields or the quality of most cash crops and can leak to surface or sub-surface waters. In some cases salinity can be associated with unique history, rarity, or special habitats protected by environmental laws. Yet in considering the measurement of soil salinity for long-term monitoring purposes, adequate methods are required. Both saturated paste extracts, intended for agriculture, and direct surface and/or porewater salinity measurement, used in inundated wetlands, are unsuited for hypersaline wetlands that often are only occasionally inundated. For these cases, we propose the use of 1:5 soil/water (weight/weight) extracts as the standard for expressing the electrical conductivity (EC) of such soils and for further salt determinations. We also propose checking for ion-pairing with a 1:10 or more diluted extract in hypersaline soils. As an illustration, we apply the two-dilutions approach to a set of 359 soil samples from saline wetlands ranging in ECe from 2.3 dS m^-1^ to 183.0 dS m^-1^. This easy procedure will be useful in survey campaigns and in the monitoring of soil salt content.

## Introduction

Salts commonly occur across the Earth’s surface. Most salts needed for life are imbibed by plants from the soil. Thus, fertile soil provides an adequate content of salts needed by plants. Contrariwise, highly soluble salts are biocides at high concentrations. Few organisms, called extremophiles, are adapted to hypersaline conditions.

Soil salts at the Earth surface are dissolved and redistributed across the landscape, leading either to salt leaching or accumulation at specific geomorphic positions. Saline soils are more frequent in regions where evaporation exceeds rainfall. Together with natural factors, human actions can salinize or desalinize soils, sometimes in only a few years. Examples of such include the clearing of lands in Australia, or irrigation with brackish water pervasive in some countries. In some cases, changes in soil salt contents have been measured or surmised at several temporal scales [1–4].

The conservation of saline enclaves comes up as the demands for environmental protection become more elaborate, e.g., [5], especially in arid lands as reviewed by Williams [6–7]. Many saline wetlands around the world are included in the Ramsar Convention signed by 167 countries (www.ramsar.org). Established in 1971, this convention was a milestone for the protection of wetlands from desiccation, or from agricultural, urban, or other uses. The concept of pedodiversity can be applied to saline soils, some of them specific to protected wetlands.

Experience concerning the study of saline sites and protection is much more limited than for agricultural salinity. A major challenge of managing protected saline wetlands is the establishment of conservation plans, with adequate monitoring and surveillance protocols. Often, managers cope with conflicts between saline ecosystem protection and surrounding agricultural or other land uses. Knowledge of hydrology, soils, and vegetation is needed to reach solid agreements with farmers and other stakeholders, and to design steps for preventing or dissuading the alteration of these saline habitats.

Both drainage and water input can result in natural or anthropic alterations of wetlands. Anthropic water inputs often contain suspended sediment, agrochemicals or other unwanted substances. Less studied has been the impact of the dilution of brine and soil solution by the outflows of water from conterminous irrigated lands, a frequent setting in dry climates [8]. Decreases in salinity can reduce or eliminate the halophytes and other extremophiles by favoring new and denser vegetation with invasive species. Direct appraisal of soil salinity evolution needs easy methods allowing robust and repeatable measurements for evaluating the long-term response of saline wetlands to large-scale perturbations. These methods will allow for quantitative comparisons and avoid frivolous qualifications of soils as salinized [9].

Once the soil and the hydrology of a saline wetland are known, changes in its habitats can be detected and assessed by measuring and comparing the soil salinity [10]. These data can be combined with airborne or satellite data for evaluating the conservation status of the saline wetlands [11]. Given a lack of adequate soil maps, botanical surveys can be a proxy for detecting saline wetlands, linking their occurrence and behavior with geologic features [12] and directing samplings needed to depict and monitor soil salinity across the landscape.

In many wetlands located in arid environments, salinity cannot be measured in surface or porewater because these wetlands are only intermittently inundated, in most cases with discontinuous water as shallow as 1 or 2 cm, and in some years never inundated; often the soil is at water potentials that make the extraction of porewater unpractical. Conversely, calculating the salinity of porewater from the saturation extract is arguable [13]. Moreover many wetlands are occasionally or permanently used for agriculture. At least in Europe, this is manifested as frivolous agriculture, implemented only to earn subsidies despite destroying valuable ecosystems in the process. One of the threats is the dramatic alteration by irrigation of the hydric regime and the ionic composition of the ecosystem.

Our proposal would standardize and streamline the long-term monitoring of the response of these protected ecosystems to perturbations at several scales. Filling the gap between “wetlands methods” and “agricultural methods” will allow the use of soil salinity data from both domains.

The most widespread way to express agricultural soil salinity is to prepare an extract of the saturated paste and measure its electrical conductivity (ECe), with further study of ions if desired. The objectives of this research are to: i) review some practical shortcomings of the saturated paste for long-term salinity appraisal in hypersaline soils, and (ii) present an easy and unsophisticated procedure based on extracts at two fixed soil to water ratios circumventing saturated paste preparation. We illustrate this procedure by the relationships between the ECe, EC1:5 and EC1:10 for a set of samples from hypersaline soils.

## The Extract of Saturation

The methods established by the United States Salinity Laboratory Staff [14] are used worldwide for soil salinity studies. These methods, aimed at agriculture, established the electrical conductivity of the saturated paste extract (ECe) for expressing soil salinity, with the extracts at other fixed soil to water ratios considered auxiliary or less important. The amount of water needed to prepare the saturated paste is reported as cm^3^ of water per 100 g of soil, i.e., weight to weight (w/w). This amount, i.e., the saturation percentage (SP), depends on the textural composition of the soil, clay mineralogy, and organic matter contents. Saturated paste was intended to enable soil solution to be extracted with simple equipment, overcoming the practical impossibility of extracting the actual soil solution used by crop roots. ECe has become a standard measure of soil salinity, widely used to compare the salt tolerance of cultivated plants, and for irrigation and drainage engineering.

The purpose of soil salinity monitoring in hypersaline soils of protected wetlands is not to determine the effects of dissolved salts on plant growth, as is the case in agricultural or in plant physiology research. The monitoring of protected wetlands will watch and ward the salt contents associated with the prevalence of the halophytes and other valuable organisms adapted to hypersalinity. The available methods to measure the electrical conductivity (EC) in-situ are influenced by temperature and soil moisture, and are unpractical in these intermittently inundated wetlands. Measures of soil salinity by a lumped parameter, EC, at standard and repeatable conditions of temperature and dilution will be needed for years to come.

## Justification for Replacing the Saturation Extract in Hypersaline Soils

### 3.1

Extracts at fixed soil to water ratios, with 1:5 and 1:10 as the more popular, have been used in soil science. The disclosure of the relationships of their EC with ECe [15] has garnered the attention of many scientists using different approaches as shown by [16]. However, the United States Salinity Laboratory Staff ([14], page 13) recommended fixed ratios for determining the change in salinity with time; in the same way, [17] recommend 1:10 for evaluation of the total soluble salts of soil and for the assessment of reclamation procedures.

The content of soluble ions is related with EC. However, at high saline concentrations, some ions of opposite charge can be bonded together forming ion-pairs with 0 or other charges [18, 19]. If ion-pairing occurs in the extracts as is the case of hypersaline soils, a specific equation linking EC with the ionic content in the extract must be established based either on chemical [18, 20–22], or statistical [23–34] procedures.

The search for such an equation can be unpractical for soil survey or monitoring operations due to the need for either supplementary data to be included in the equation or chemical considerations often applied with ionic speciation software. However, if the interest is focused on the off-site effects of soil salinity (i.e., salt discharge to surface and underground waters, rather than on the effects of salinity on plants), a fixed soil to water ratio more diluted than the saturation extract can better estimate the content of salts in soils.

### 3.2

The non-occurrence of ion-pairing is a condition to have a simple relationship of EC with the content of salts in solution. If ion-pairing occurs, the measure of the salt content would require either gravimetry of the total dissolved salts or the individual titration of soluble ions. Ion-pairing does not occur in saturation extracts of low EC; e.g., in the experiments of [35] with a non-saline soil of ECe = 0.66 dS m^-1^. However, the reservations [36–37] for soil salinity estimates from ECe in non-saline calcareous soils can be extended to gypsiferous soils [29].

### 3.3

The correspondence between ECe and salt content diminishes at high salinities, with the classical olifant-shaped silhouette in the scatter diagrams of ions vs. ECe [34] needing logarithmic transformations to approach linear relationships. Many soil scientists [38, 39] have shown the problems of relating ECe with total salt content for hypersaline soils. Similar concerns were expressed by [40] for ECe > 8 dS m-1 when relating ECe with the electrical conductivity measured in the saturated paste. Moreover, [36] found that for non-saline calcareous soils, saturation extracts require dilution by a factor of 1000 to accurately predict soil salinity. The aforementioned considerations jeopardize the meaning of ECe and also the additivity of this magnitude from different samples, and can question the meaning of averaged ECe for pedons sampled at several depths, or for multiple samples when estimating soil salinity of broad extensions. These constraints can be dramatically reduced if no ion-pairing happens, as would be the case for extracts more diluted than those from saturated paste.

### 3.4

Most ECe measurements and subsequent determinations in the saturation extracts are used for agriculture or for plant salt-tolerance appraisal e.g., [41–43], and for the study of saline habitats, e.g., [44, 45]. Most of these works do not report saturation percentage (SP). However, when ECe is to be used for comparison of salinity in the same soil, evaluation of the deviations in SP between different times or laboratories [27, 34, 46] is compulsory. Yet often SP is not reported in comparisons of salinity, e.g., [2, 47, 48]. A 5% SP deviation has been suggested as an allowable difference ([49], page 265). This is a key issue for comparisons involving different technicians or laboratories, especially for long-term monitoring. The measurement of changes in ECe and ion concentrations can be biased not only because of the different criteria in the end-point of the paste but also by the grinding or extraction methods [50–51].

Moreover while centrifugation of the saturated paste is used by many labs for obtaining the extract, many others use vacuum extraction, a technique that concentrates the extract, as evidenced by the descent of temperatures in the flasks produced by evaporation under vacuum. In practice, extract concentration achieved by evaporation cannot be compensated by calculations, and will variably affect the extraction depending on the intensity and time of vacuum, lab temperature, or the whole volume of air evacuated by the pump. The total effects will be associated with the granulometry and other properties of the soil sample, affecting each sample at different degrees even within the same extraction set.

### 3.5

The saturation extract cannot be obtained in field labs. Moreover, saturated paste preparation is made by hand, with no prospects for automatization, and the extraction of the solution by vacuum can last more than one hour. The process is lengthy and hard to perform under the current conditions of dismantlement of many soil labs. This, plus the scarcity of technicians trained in the preparation of saturation extracts endorses the use of extracts at fixed water to soil ratios for surveys or other works of great spatial or temporal span needing serialized determinations.

### 3.6

Natural hypersaline soils occur both in the coasts and inland with salinity levels and other circumstances precluding the growth of common profitable crops. Moreover environmental regulations of some countries forestall agriculture in these wetlands. Very often, the appraisal of soil salinity by EC in such environments is needed for applications other than agriculture. Most of these soils are intermittently inundated, or at least saturated, by salty water or brine. For salt-tolerance evaluation, it seems trivial to relate ECe or EC at other dilution ratios with the natural composition of the soil solution that would limit the growth of plants. Under these conditions, to appraise the content of salts is more interesting than to approach the salinity affecting plant growth as intended by the saturated paste method. Thus, application of a fixed ratio should be better than saturation extracts.

### 3.7

Very often, the saturation extracts from saline wetlands make ion-pairs, while in most cases the 1:5 and 1:10 extracts will not do that. Thus, the relationships between the ionic concentration and EC will be simpler than the saturation extract. A noteworthy advantage of these extracts for hypersaline soils would be to start in the lab from concentrations more convivial with the conventional chemistry labs titrating ions with methods or equipment not devised for high ionic concentrations. It will reduce or eliminate the need for time consuming dilutions; a classical source of mistakes and errors in serial analytical determinations.

### 3.8

Extracts at 1:5, 1:1, or other fixed soil to water ratios are often used in different scientific domains [52–77]. Regrettably, sometimes the dilution ratio is missing or not clearly stated in reports or articles [78–81] or maybe this ratio, saturation or other, is assumed, e.g., [82–86], compromising or precluding future comparisons and generalization. As saturated paste is unlikely to be adopted as a standard in many of these scientific domains, and the SP is very often not reported, fixed soil to water ratios would be easier and more repeatable. In short, should the methods of soil science depart from other scientific disciplines like biology or ecology that also conduct studies on soil salinity?

### 3.9

Even though in our experience no ion pairing happens in 1:5 extracts, this circumstance merits checking in hypersaline soils. The method of diluting the saturation extract until EC reaches a value between 0.1–0.3 dS m^-1^ [87] overcomes ion-pairing to achieve a linear relationship with the salt content of the saturated extract. This method could be used for extracts at fixed ratios (e.g., 1:5), where the occurrence of ion-pairing was suspected, but regression with a lower ratio extract (e.g., 1:10) is easier and ancillary to a duplicate determination of the EC1:5. [88] successfully used paired 1:5 and 1:10 soil to water extracts in seven saline wetlands. Should the relationships between EC1:5 and EC1:10 depart from the linearity pointing to ion-pairing, an extraction at more diluted ratios would be needed.

## The Two Dilutions Extract Approach

Our proposal for soil salinity expression for monitoring purposes is to prepare both 1:5 and 1:10 extracts. The regression between EC1:5 and EC1:10 will be a check for the absence of ionic pairs. This easy procedure is free of chemical models and permits a choice of the extract to be used for further titration of ions. The second extracts can be considered as surrogates for duplicated determinations. Depending on the desired confidence level, the second dilution can be limited to a reduced subset of soil samples if only a statistical check of no ionic-pairing is desired.

The effects of the preparation procedures of the 1:5 extract using a set of 20 samples ranging in ECe from 0.96 to 21.20 dS m^-1^, and in SP from 32.4% to 68.1%, were studied by [89]. After selecting the appropriate procedure, they stressed the need to report its detailed description.

Our approach involving two different soil to water ratios can be transposed to the methods based on volume ratio extracts instead of weight ratios, as proposed by [90] for glasshouse soils. More recently, upon the establishment of the suborders Wassents and Wassists in US Soil Taxonomy [48], a method for measuring the EC of subaqueous soils has been defined by Soil Survey Staff ([91], page 292). This method measures the EC from a fresh, field wet sample using a mixture (not extract) of soil to water at the ratio of 1:5 by volume (EC1:5 vol), instead of the conventional 1:5 ratio by weight, and measures the EC of the supernatant. If, for taxonomic or characterization purposes the salinity is to be calculated from EC1:5 vol, the eventual occurrence of ion-pairing could be checked with the extract of 1:10 vol.

## Dual Dilution Methodology for Hypersaline Soils

### 5.1. Materials and methods

**Fig 1:**
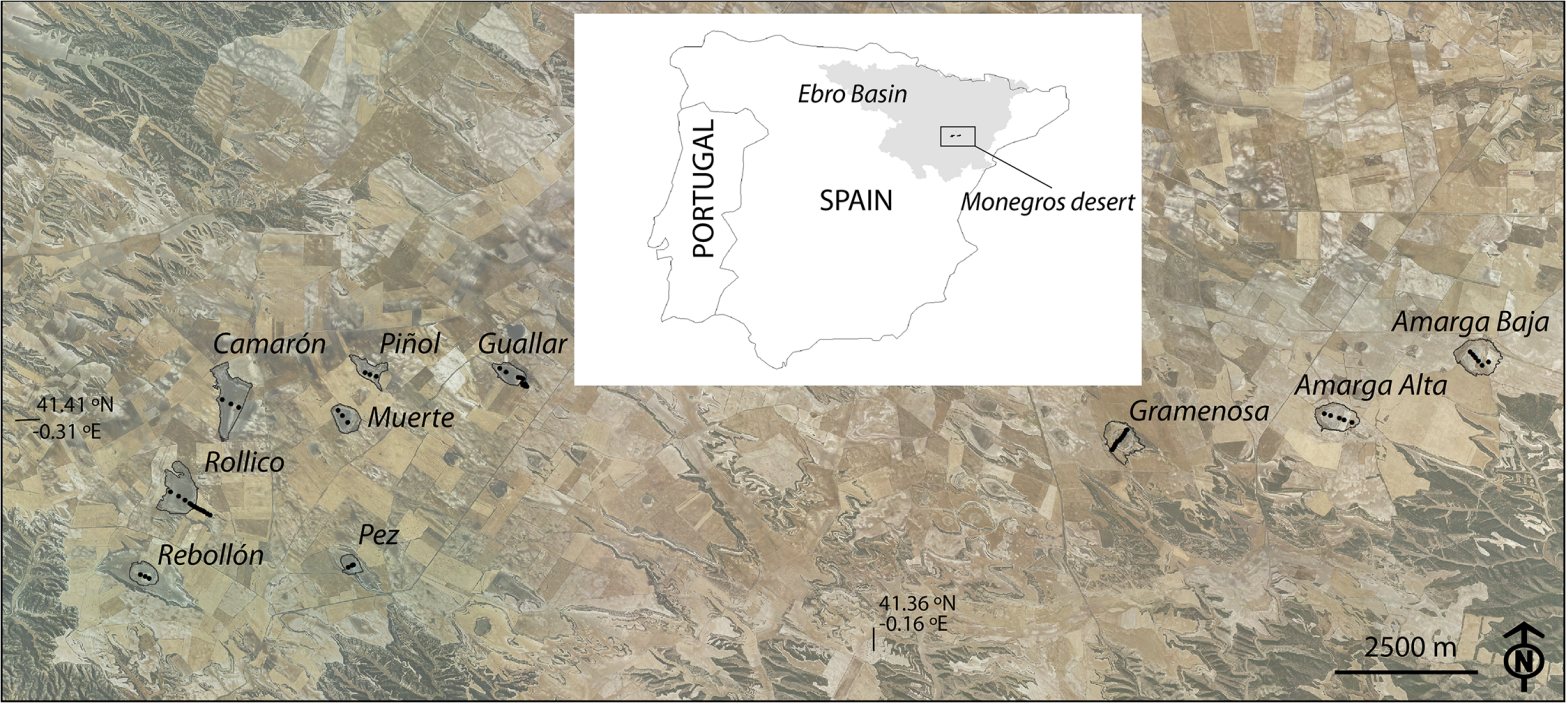
Soil sampling sites marked with dots on an orthophoto (PNOA, Instituto Geográfico Nacional) taken in 2009. UTM coordinates are listed at S1 Table. The insert shows the location of the Monegros area within the Ebro Basin, Spain.

We studied several hypersaline wetland “saladas” protected under Spanish environmental regulations and located in the Monegros desert, Spain (Fig 1). The geological framework of the distribution of these wetlands was addressed by [12]. The soils of the saladas were Gypsic Aquisalids and Typic Haplogypsids per US Soil Taxonomy [49]. Permissions for sampling were granted by the Authority in charge of protected areas: Instituto Aragonés de Gestión Ambiental (www.aragon.es/inaga). The soils at the floors of ten of these saladas: Amarga Alta, Amarga Baja, Camarón, Gramenosa, Guallar, Muerte, Pez, Piñol, Rebollón, and Rollico (Fig 1), were hand-augered at 59 sites, taking cores by depth increments of 20–25 cm. The resulting 359 soil samples were air-dried first at room temperature and then in a ventilated oven for 2 weeks at 40°C in order to avoid the destruction of gypsum (CaSO_4_•2H_2_O). Samples were ground to pass a 2-mm sieve; no coarse fragments occurred. The saturated pastes were prepared with their saturation percentage (SP) recorded. The electrical conductivity (EC) was measured from the saturation extract (ECe) and from extracts with soil to water ratios in weights of 1:5 (EC1:5) and 1:10 (EC1:10). All the electrical conductivities were expressed in dS m^-1^ at 25°C.

All samples contained gypsum, a common mineral in saline wetlands. Then, sufficient time was allowed to guarantee the maximum dissolution of gypsum to attain stable and reproducible EC measurements. The saturated paste was left to stand overnight before extraction. For the 1:5 and 1:10 extracts, we applied 30 min of reciprocal shaking at 175 oscillations per minute followed by overnight standing before filtration for EC measurement.

The Cl^-^ was titrated in 288 samples from eight of the studied saladas on extracts at 1:10 dilution using a potentiometer, and expressed in milliequivalents per liter (meq L^-1^). Gypsum content was determined by thermogravimetry per [92] and calcium carbonate equivalent was measured by gasometry with a Bernard calcimeter.

After exploratory data analysis of the data shown at S2 Table, ordinary least squares (OLS) regression was applied with the coefficient of determination (R^2^, expressed as %) and the standard error (SE) calculated.

### 5.2. Results

**Fig 2:**
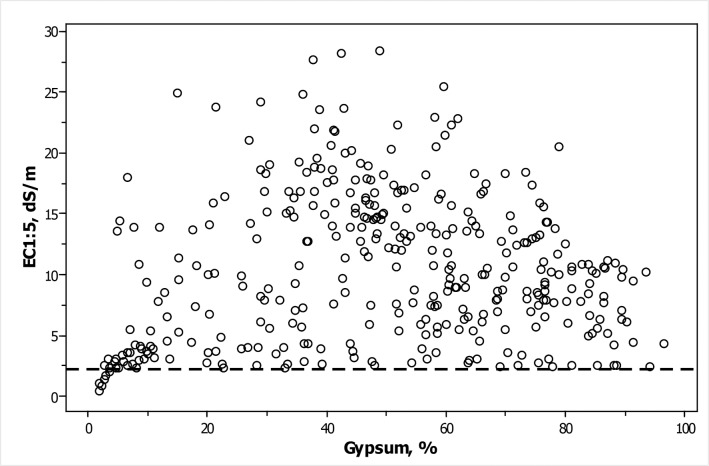
Scatterplot of EC1:5 on the gypsum content for 356 soil samples from Spain having both determinations. Dashed line marks 2.25 dS m^-1^.

The sampled soils were either bare or supported halophytes at the less inundable positions. This is in agreement with the hypersalinity of the studied soils expressed by the mean ECe = 72.3 dS m^-1^ of the 359 samples studied and their range from 2.32 dS m^-1^ to 183.00 dS m^-1^ (Table 1). Ten samples were below the ECe threshold 4 dS m^-1^ for saline soils, while 300 samples had ECe > 16 dS m^-1^, the threshold for very strongly saline soils. Only 14 samples had EC1:10 ≤ 2.25 dS m^-1^, the approximate electrical conductivity produced by calcium sulfate saturation marked with a dashed line in Fig 2.

**Table 1:** Descriptive statistics from the determinations in the *N* soil samples analyzed.

Unit	Determination	*N*	Mean	Min.	Max.
%	SP	359	39.07	21.0	68.0
dS m^-1^	ECe	359	72.28	2.32	183.00
	EC1:5	359	10.50	0.41	28.40
	EC1:10	359	6.58	0.33	16.30
meq L^-1^	Cl1:10	288	42.32	0.40	131.7
%	Gypsum	356	49.32	1.87	96.44
	CCE	356	22.51	0.48	72.65
	Gypsum + CCE	356	71.83	37.83	99.58

**Fig 3:**
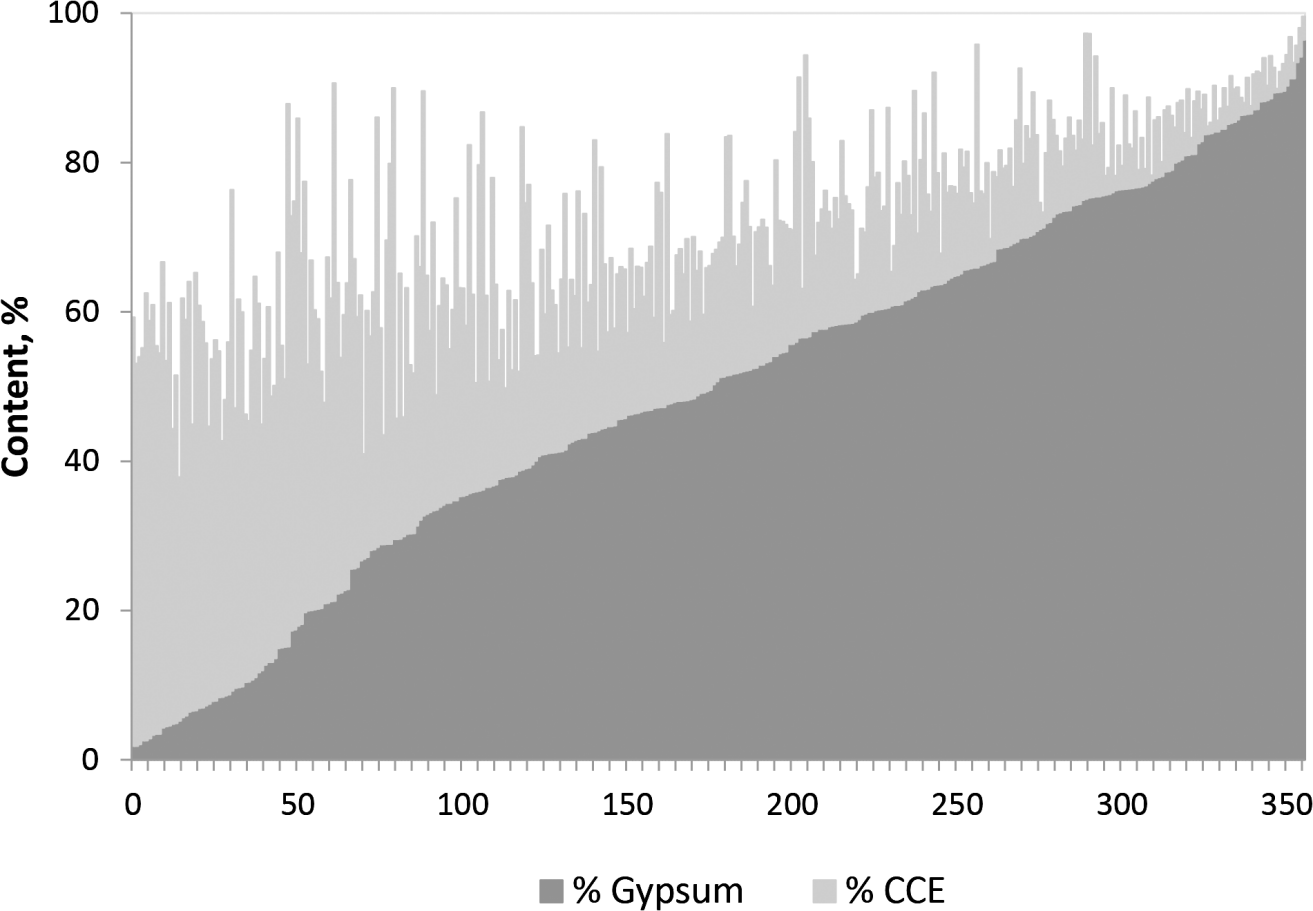
Contents of gypsum and calcium carbonate equivalent (CCE) in 356 soil samples from Spain having both determinations. Samples are ranked by their gypsum content.

Table 1 shows the high contents of gypsum and calcium carbonate. The gypsum content ranged from 1.9% to 96.4%, with a mean of 49.3%. Fig 3 shows the samples ranked by gypsum content, with 180 samples having > 50% gypsum. Only three samples with gypsum ranging from 1.9% to 2.1% had their gypsum content below the thresholds for saturating the 1:5 or the 1:10 dilutions, i.e., 1.2% and 2.4% gypsum, respectively, assuming that saturation is attained at 2.4 g of gypsum per liter of pure water, a solubility that increases if Cl^-^ or other non-common ions are present. Most samples qualify as gypseous [93], but their high salinity supersedes other limitations to life [94].

Fig 3 also shows the contents of calcium carbonate equivalent (CCE). The sum of CCE plus gypsum ranged from 37.8% to 99.6%, with a mean of 71.2%; this sum was > 50% for 338 samples of the 356 having both gypsum and CCE titrated. The average SP = 39.1% falls within the range of the SP reported for coarse or sandy textures [14, 95]. It agrees with the field textures of these soils, controlled by the abundance of visible-sized gypsum crystals.

**Fig 4:**
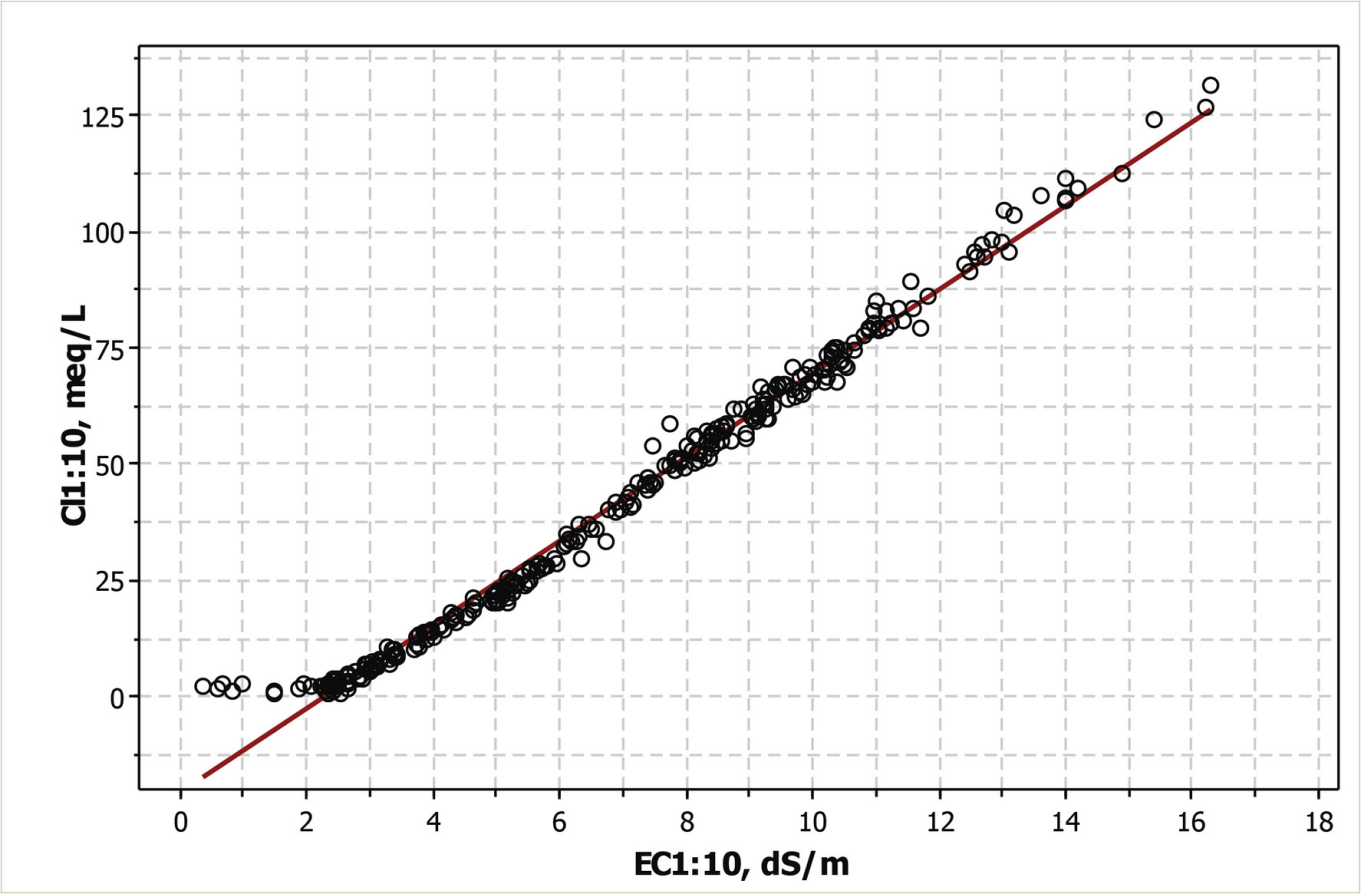
Scatterplot of the Cl- concentration on the EC, both determined at the 1:10 soil to water extracts.

The Cl^-^ concentration (Cl1:10) in the 1:10 extracts of the 288 samples analyzed showed a linear distribution against the EC of the same extracts, in spite of the inflection in the scatterplot at EC ~2.25 dS m^-1^ (Fig 4).

The OLS adjustment is given in Eq 1:

Cl1:10=−20.5+9.00×EC1:10 (Eq 1)

with R^2^ = 98.8% and SE = 3.42 meq L^-1^

If only the samples with EC1:10 > 2.25 dS m^-1^ are computed, the OLS adjustment for the 276 remaining samples is given in Eq 1A:

Cl1:10=−22.7+9.25×EC1:10 (Eq 1A)

with R^2^ = 99.4% and SE = 2.33 meq L^-1^

**Fig 5:**
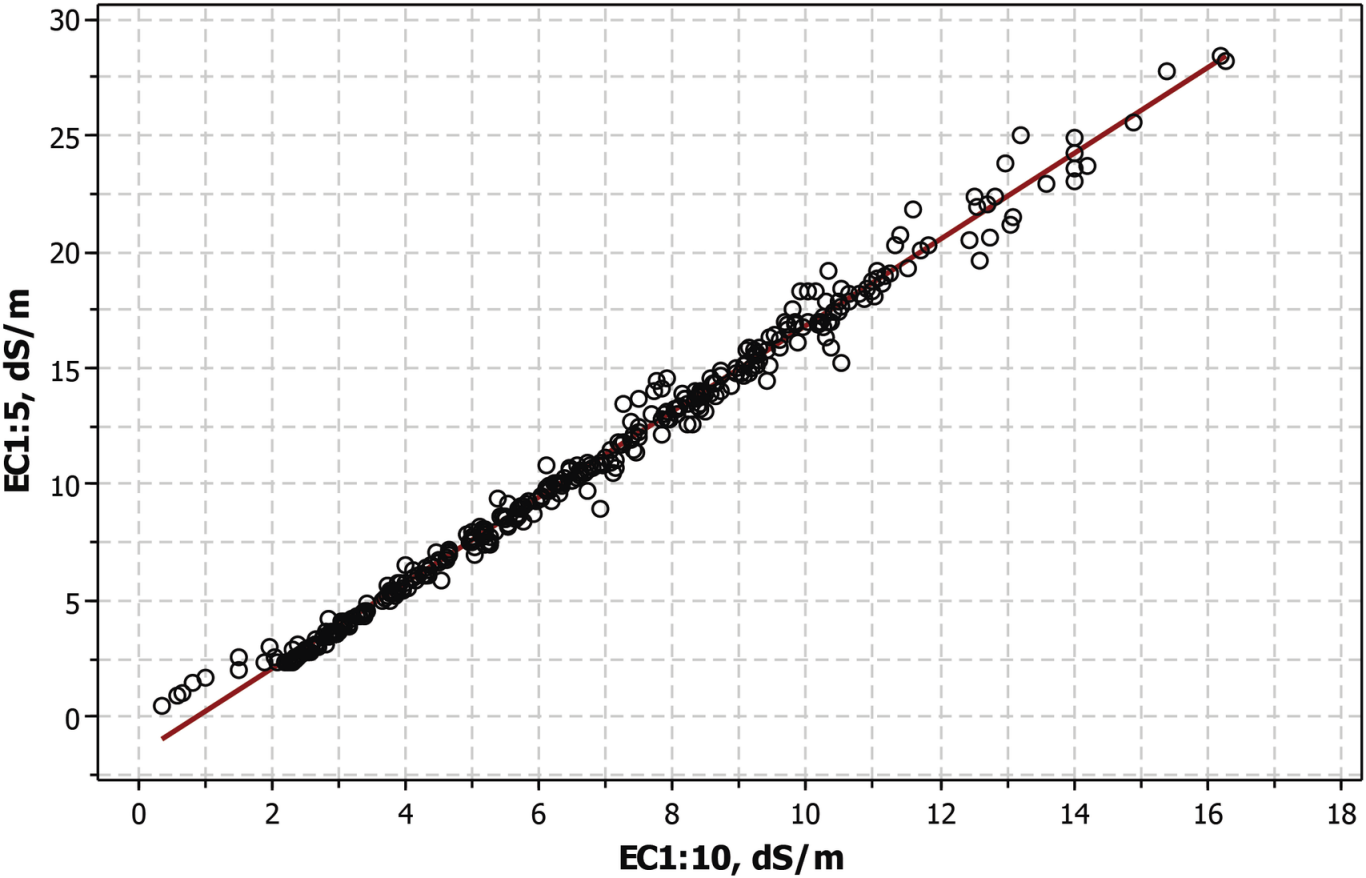
Scatterplot of EC1:5 on EC1:10 and straight line adjusted by OLS regression. The presence of gypsum is associated with the inflection in the distribution occurring at EC values around 2.25 dS m^-1^.

The relationship between the EC at the two soil to water dilutions of 1:5 and 1:10 was studied by scatterplot (Fig 5) and by OLS regression using the 359 soil samples.

The OLS adjustment is given in Eq 2:

Cl1:5=−1.57+1.84×EC1:10 (Eq 2)

with R^2^ = 99.1% and SE = 0.57 dS m^-1^

If only the samples with EC1:10 > 2.25 dS m^-1^ are computed, the OLS adjustment for the 345 remaining samples is given in Eq 2A:

EC1:5=−1.73+7.85×EC1:10 (Eq 2A)

with R^2^ = 99.2% and SE = 0.54 dS m^-1^.

## Discussion

We have reviewed the shortcomings of ECe for the study of soil salinity in hypersaline environments with no agricultural purposes, and the advantages of using more diluted extracts. Our proposal of assessment of salinity is based on two extractions at different soil to water ratios, 1:5 and 1:10. The preparation of these extracts is much easier, less time-consuming, and equipment-demanding than saturation extracts. Also, further titration of individual ions would be easier than from the saturation extract, which is much more concentrated. Another advantage of the 1:5 extract is the coincidence with the common practice in Australia of expressing salinity by EC1:5 [31, 96] and by the Cl^-^ concentration in this extract [97].

The quality of the analyses in our example is supported by the scatterplot of Cl^-^ on EC in the 1:10 extracts (Fig 4) and by the linear relationship between these determinations shown by Eq 1 with R^2^ = 98.8%. The same support is provided by the scatterplot of EC1:5 on EC1:10 (Fig 5) and by the R^2^ = 99.1% attained by the OLS regression (Eq 2). Straight line adjustments indicate that both extracts have the same degree of ionization, i.e., no ion-pairing happens. Systematic departures from the adjusted lines occur for the few soil samples which lack sufficient gypsum or other soluble minerals for achieving an EC1:10 of 2.25 dS m^-1^. If these samples are eliminated from the adjustments (Eqs 1A and 2A) the slight improvements are below the average allowable errors of most routine lab analyses.

The ubiquity of gypsum in the studied samples, a frequent setting in athalassohaline wetlands, and their specific ionic composition prevents comparisons of the above adjustments with other soils. The purpose of the adjustments was not predictive, but a check of both the full ionization in the 1:5 dilution and the analytical quality. Thus, depending on circumstances like the confidence in the lab performance, the allowable lab workload, and the number of samples, the determinations of EC1:10 could be limited to a reduced number of samples covering all range of salinity determined at 1:5 dilution.

The scatterplot of EC1:5 over EC1:10 (Fig 5) shows the classical inflection due to gypsum. The samples having either EC1:5 or EC1:10 ≤ 2.25 dS m^-1^ are located under the inflection in the figure, showing in this region a systematic departure from the OLS regression line.

The presence of gypsum was early recognized as a source of troubles in the conversion of EC between extracts at different soil to water ratios ([98], page 14). That notwithstanding, the effect of gypsum producing an EC of ~2.25 dS m^-1^ at saturation, can be neglected in these conversions for hypersaline soils if the gypsiferous and non-saline samples are a minority. These samples, grouping around the point ECe = EC1:5 = 2.2 dS m^-1^ [99], can compromise the homocedasticity of the distribution if they are numerous.

If a significant number of extracts with EC < 2.25 dS m^-1^ occurs, or if specific consideration is wanted for these extracts, a separate regression could be undertaken. However, categorizing soils into groups with or without any measurable amount of gypsum to improve the prediction of ECe [29] with a separate determination of gypsum would be cumbersome for long series of determinations and for simple labs. A similar procedure based on a qualitative assessment of gypsum by precipitation with acetone in 410 soil extracts at 1:5 dilution was used in gypseous soils by [99].

Several authors have used EC1:5 as an auxiliary variable for soil mapping with electromagnetic induction (EMI) measurements [100], or have calibrated EMI measurements with EC1:5, as is the case of [52, 101–102] and the examples mentioned by [103]. In our experience, the calibration of EMI measurements with EC1:5 was possible, even if the attained coefficients of determination were lower than with ECe [99, 104]. EMI devices, as other sensors, respond to both variable and invariable soil characteristics and their distribution along the soil profile such as moisture, temperature, mineralogy, pore size and architecture, etc. Therefore, it is requisite to take some soil samples for calibrating the sensor signal with the target characteristic. EMI has been used to map soil salinity in areas with shallow water tables in the central Ebro valley, e.g. [105–107], and in saline coastal wetlands, e.g. [33, 108, 109]. The adoption of EC1:5 as the standard measure of soil salinity would simplify the calibrations and eliminate the time consuming preparation of the saturation extract.

Other developing technologies will be able to measure salinity in the field. One example is portable x-ray fluorescence spectrometry whereby Cl^-^ or other elements are used as a proxy for soil salinity, but it also requires correction for soil moisture when levels are above 20% [110, 111]. It shows promise for soil salinity appraisal, and could overcome the measurements of ECe on water extracts. These matters fall out of the scope of this article and merit discussion and effort both for comparisons and for long term monitoring of soil salinity.

**Fig 6:**
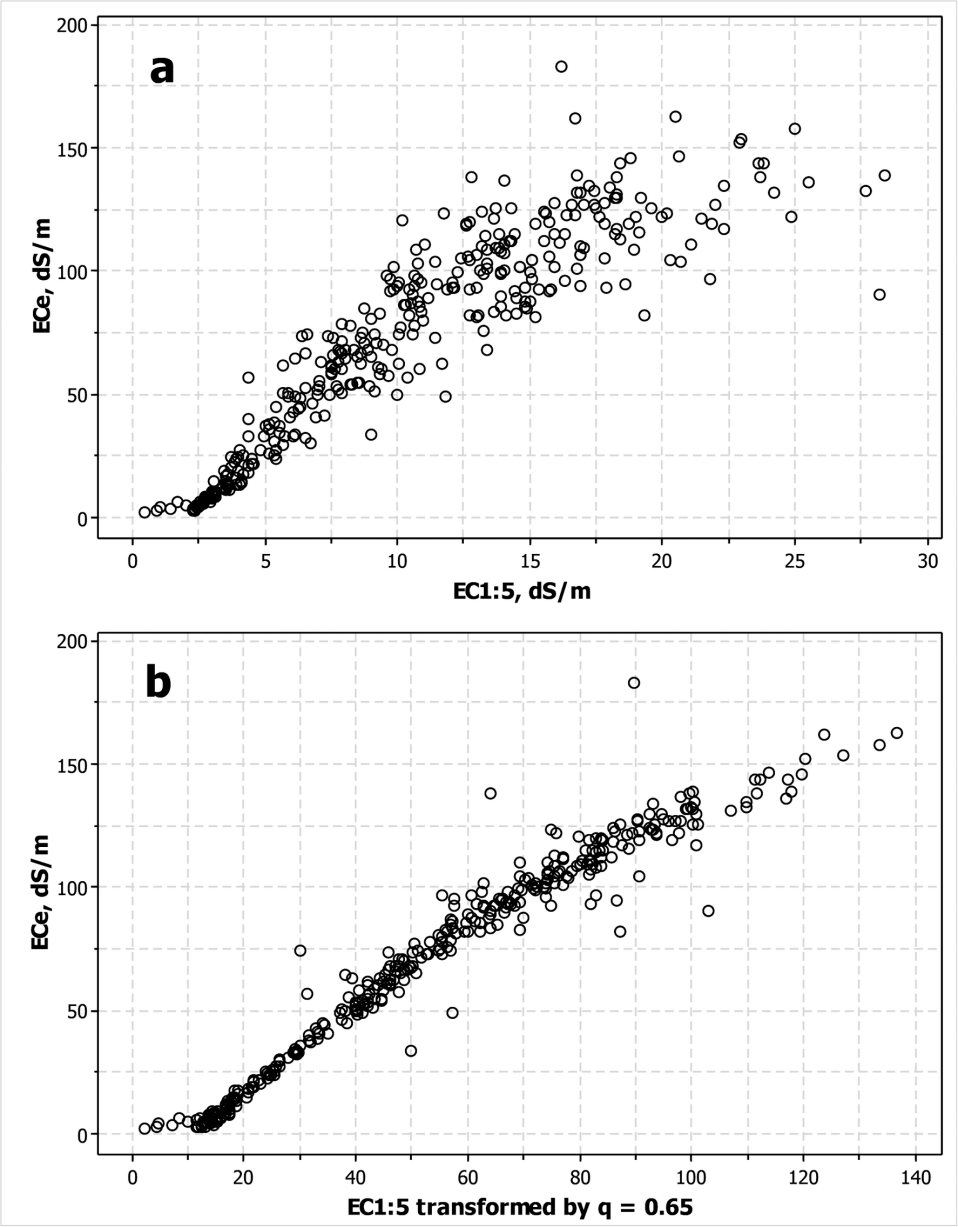
Scatterplot of ECe on: a) EC 1:5, and b) EC1:5 transformed by multiplying by (500/SP)^q^, i.e., the quotient of the dilutions of both extracts to the empirical power q = 0.65.

The multitude of models and adjustments proposed in the literature for predicting ECe from EC at a fixed soil to water ratio do not provide a conclusive, universal, and non-sophisticated procedure. For the studied soils, Fig 6A shows the inflection due to gypsum plus the classical dispersion at higher values; both features entangling the response model. For large sets of samples, one can undertake an adjustment of EC1:5 or other fixed ratios versus a reduced number of ECe determinations. However, it must be noted that the “gypsum inflection” lasts (Fig 6B) even after the transformation that includes SP [46, 112] and enables for decreasing the dispersion. Moreover, the shortcomings mentioned at Sections 3.3 and 3.4 strongly question the estimation of total salt content from ECe, at least for soils containing calcium carbonate or gypsum as well as for hypersaline soils.

## Conclusions

The study of inland wetlands located in arid climates requires the development of new concepts to be incorporated into mainstream wetland science. With the proposed approaches, we try to simultaneously assess “agricultural” and “environmental” methods that are studying the same natural objects, saline wetlands.

The degrees of salinity of the studied soils plus frequent flooding preclude their agricultural use. This setting is frequent in saline wetlands around the world that are often protected by environmental rules. Conservation of these valuable habitats requires monitoring the salt contents of the soils, diminishing the usefulness and meaning of saturated paste. The expression of total salt content by the electrical conductivity in the extracts at 1:5 dilution, with checking for no ion-pairing with the 1:10 extract, has shown to be easy, unsophisticated, and robust. Our two dilution approach bypasses the agricultural salinity expression by saturation extract by adopting the 1:5 extract as a standard for soil salinity expression instead of the saturation extract.

## Supporting Information

S1 Table UTM coordinates (European Datum ED 50) of the 59 sampling sites with their vegetation and the depth of the augerings. (PDF)

S2 Table Analytical data of the soil samples. (PDF)

## References

[1] Amundson RG, Lund LJ. Changes in the chemical and physical properties of a reclaimed saline-sodic soil in the San Joaquín Valley of California. Soil Science. 1985; 140: 213–222.

[2] Hao X, Chang C. Does the long-term heavy cattle manure application increase salinity of a clay loam soil in semi-arid southern Alberta? Agriculture, Ecosystems and Environment. 2003; 94: 89–103.

[3] Wu J, Li P, Qian H, Fang Y. Assessment of soil salinization based on a low-cost method and its influencing factors in semi-arid agricultural area. Environmental Earth Science. 2014; 71: 3465–3475.

[4] Xu Y, Pu L, Zhu M, Li J, Zhang M, Li P, et al Spatial variation of soils salinity in the Coastal Reclamation Area, Eastern China. Journal of Coastal Research. 2014; 30: 411–417.

[5] Kortekaas KH. Sustainable tourism initiatives in European saltscapes. In: Pineda FD, Brebbia CA, editors. Sustainable Tourism, WIT Transactions on Ecology and the Environment; 2004. pp.199–207.

[6] Williams WD. Conservation of wetlands in drylands: a key global issue. Aquatic Conservation: Marine and Freshwater Ecosystems. 1999; 9: 517–522.

[7] Williams WD. Environmental threats to salt lakes and the likely status of inland saline ecosystems in 2025. Environmental Conservation. 2002; 29: 154–167.

[8] Gutiérrez-Cánovas C, Velasco J, Millán A. Effects of dilution stress on the functioning of a saline Mediterranean stream. Hydrobiologia. 2009; 619: 119–132.

[9] Herrero J. La salinidad de suelos y su apreciación bifronte. In: Rubio JL, Ferri AM, editors. Medio ambiente, un medio de oportunidades. 17^th^ Foro Universitario Vives. Vol. 2. Valencia, Spain; 2009. pp. 346–354.

[10] Fowler D. Evaluating abiotic influences on soil salinity of inland managed wetlands and agricultural fields in a semi-arid environment. M.Sc. Thesis. Louisiana State University. 2013.

[11] Castañeda C, Herrero J. Assessing the degradation of saline wetlands in an arid agricultural region in Spain. Catena. 2008; 72: 205–213.

[12] Castañeda C, Herrero J, Conesa JA. Distribution, morphology and habitats of saline wetlands: A case study from Monegros. Geologica Acta. 2013; 11: 371–388.

[13] Watson EB, Byrne R. Abundance and diversity of tidal marsh plants along the salinity gradient of the San Francisco Estuary: implications for global change ecology. Plant Ecology. 2009; 205: 113–128.

[14] United States Salinity Laboratory Staff. Diagnosis and improvement of saline and alkali soils Agriculture Handbook no. 60 USDA; 1954. Reprinted 1969.

[15] Paliwal KV, Maliwal GL, Yadav BR. Prediction of soil salinity at saturation by different soil-wter ratios. Current Agriculture. 1978; 2: 43–47.

[16] Sonmez S, Buyuktas D, Okturen F, Citaj S. Assessment of different soil to water ratios (1:1, 1:2.5, 1:5) in soil salinity studies. Geoderma. 2008; 144: 361–369.

[17] Chang C, Sommerfeldt TG, Carefoot JM, Schaalje GB. Relationships of electrical conductivity with total dissolved salts and cation concentration of sulfate-dominant soil extracts. Canadian Journal of Soil Science. 1983; 63: 79–86.

[18] Tanji KK. Predicting specific conductance from electrolytic properties and ion association in some aqueous solutions. Soil Science Society of America Proceedings. 1969; 33: 887–890.

[19] Darab K, Csillag J, Pinter I. Studies on the ion composition of salt solutions and of saturation extracts of salt-affected soils. Geoderma. 1980; 23: 85–111.

[20] Adams F. Ionic concentration and activities in soil solution. Soil Science Society of America Proceedings. 1971; 35: 420–426.

[21] Visconti F, de Paz M, Rubio JL. What information does the electrical conductivity of soil water extracts of 1 to 5 ratio (w/v) provide for soil salinity assessment of agricultural irrigated lands? Geoderma. 2010; 154: 387–397.

[22] Visconti F, de Paz M, Rubio JL. An empirical equation to calculate soil solution electrical conductivity at 25 degrees C from major ion concentrations. European Journal of Soil Science. 2010; 61: 980–993.

[23] Richard M, Gouny P. Contrôle de la salinité des sols. Annales agronomiques. 1965; 16: 625–635.

[24] Pérez-García V, Fernández-Caldas E, García V. Distribución y características de los suelos salinos de la Isla de Tenerife. Anales de Edafología y Agrobiología. 1975; 34: 595–565.

[25] Le Brusq JY, Loyer JV. Relations entre les mesures de conductivités sur des extracts de sols de rapports sol/solution variables dans la vallée del fleuve Senégal. Cahiers ORSTOM, série Pédologie. 1982; 19: 293–301.

[26] Hogg TJ, Henry JL. Comparison of 1:1 and 1:2 suspensions and extracts with the saturation extract in estimating salinity in Saskatchewan soils. Canadian Journal of Soil Science. 1984; 64: 699–704.

[27] Herrero J, Pérez-Coveta O. Soil salinity changes over 24 years in a Mediterranean irrigated district. Geoderma. 2005; 125: 287–308.

[28] Khorsandi F, Yazdi FA. Gypsum and texture effects on the estimation of saturated paste electrical conductivity by two extraction methods. Communications in Soil Science and Plant Analysis. 2007; 38: 1105–1117.

[29] Khorsandi F, Yazdi FA. Estimation of saturated paste extracts' electrical conductivity from 1:5 soil/water suspension and gypsum. Communications in Soil Science and Plant Analysis. 2011; 42: 315–321.

[30] Chi CM, Wang ZC. Characterizing salt-affected soils of Songnen Plain using saturated paste and 1:5 soil-to-water extraction methods. Arid Land Research and Management. 2010; 24: 1–11.

[31] He YB, DeSutter T, Prunty L, Hopkins D, Jia XH, Wysocki DA. Predicting ECe of the saturated paste extract from value of EC1:5. Canadian Journal of Soil Science. 2013; 93: 585–594.

[32] Bilgili AV, Aydemir A, Sönmez O, Çullu MA. Comparison of three laboratory and one regression kriging method for quantitative and qualitative assessment of soil salinity in the Harran plain, SE Turkey. Fresenius Environmental Bulletin. 2013; 22: 1339–1350.

[33] Guo Y, Shi Z, Li HY, Triantafilis J. Application of digital mapping methods for identifying salinity management classes based on a study on coastal central China. Soil Use and Management. 2013; 29: 445–456.

[34] Herrero J, Castañeda C. Changes in soil salinity in the habitats of five halophytes after 20 years. Catena. 2013; 109: 58–71.

[35] De Souza ER, De Melo HF, Almeida BG, Melo DVM. Comparison of methods for extracting soil solution. Revista Brasileira de Engenharia Agricola e Ambiental. 2013; 17: 510–517.

[36] Amakor XN, Jacobson AR, Cardon GN. Improving estimates of soil salinity from saturation paste extracts in calcareous soils. Soil Science Society of America Journal. 2013; 77: 792–799.

[37] Semiz GD, Atmaca Ö. Determination of regression equations to predict soil extract salinity from aquaeous soil samples (1:2 and 1:5) for calcareous and non-calcareous loamy soils. Journal of Food, Agriculture & Environment. 2013; 11: 748–750.

[38] Álvarez-Rogel J, Hernández J, Ortiz-Silla R, Alcaraz F. Patterns of spatial and temporal variations in soil salinity: example of a salt marsh in a semiarid climate. Arid Soil Research and Rehabilitation. 1997; 11: 315–329.

[39] Shahid SA, Abdelfattah MA, Mahmoudi H. Innovations in soil chemical analyses: New ECs and total salts relationship for Abu Dhabi Emirate soils In: Shahid SA et al, editors. Developments in soil classification, land use planning and policy implications; Springer Science 2013 pp 799–812

[40] Zalba P, Garay M, Amiotti N, Ares A. Improved field method for estimating soil salinity. Ciencia del Suelo. 2013; 31: 265–269.

[41] Ulery AL, Teed JA, van Genuchten MT, Shannon MC. Saltdata: A database of plant yield response to salinity. Agronomy Journal. 1998; 90: 556–562.

[42] Maas EV, Grattan SR. Crop yields as affected by salinity In: Skaggs RV, Van Shilfgaarde J, editors. Agricultural drainage. American Society of Agronomy, Madison, WI; 1999 pp. 55–108.

[43] Díaz FJ, Benes SE, Grattan SR. Field performance of halophytic species under irrigation with saline drainage water in the San Joaquín Valley of California. Agricultural Water Management. 2013; 118: 59–69.

[44] Castroviejo S, Porta J. Apport à l’écologie de la végétation des zones salées des rives de la Gigüela, Espagne. Les vases salées, Colloques phytosociologiques de Lille. 1975; IV: 115–139.

[45] Cantero JJ, León R, Cisneros JM, Cantero A. Habitat structure and vegetation relationships in central Argentina salt marsh landscapes. Plant Ecology. 1998; 137: 79–100.

[46] Herrero J. Salinidad edáfica en varios salobrares de Aragón. Memorias de la Real Sociedad Española de Historia Natural. Tomo IV. Madrid, Spain; 2008.

[47] Li KL, Chen J, Tan MZ, Zhao BZ, Mi SX, Shi XZ. Spatio-temporal variability of soil salinity in alluvial plain of the lower reaches of the Yellow River-A Case Study. Pedosphere. 2011; 21: 793–801.

[48] Güler M, Arslan H, Cemek B, Erşahin S. Long-term changes in spatial variation of soil electrical conductivity and exchangeable sodium percentage in irrigated mesic Ustifluvents. Agricultural Water Management. 2014; 135: 1–8.

[49] Soil Survey Staff. Keys to Soil Taxonomy, 12th ed. USDA-NRCS. U.S. Gov. Print. Office, Washington DC; 2014.

[50] Jacober F, Sandoval F. Effect of soil grinding, suction, and extraction time on salt concentration of saturation extracts. Soil Science. 1971; 112: 263–266.

[51] Cooper CA, Davis JG, Cardon GE. Influence of laboratory methods on calcareous saline soils for EC measurements and leaching. Communications in Soil Science and Plant Analysis. 2008; 39: 329–343.

[52] Williams BF, Baker GC. An electromagnetic induction technique for reconnaissance surveys of salinity hazards. Australian Journal of Soil Research. 1982; 20: 107–118.

[53] Meyer SE, García-Moya E, Lagunes-Espinoza LD. Topographic and soil surface effects on gypsophile plant community patterns in central Mexico. Journal of Vegetation Science. 1992; 3: 429–438.

[54] Sylla M, Stein A, Vanbreemen N, Fresco LO. Spatial variability of soil-salinity at different scales in the mangrove rice agroecosystem in West-Africa. Agriculture Ecosystems & Environment. 1995; 54: 1–15.

[55] Shirokova Y, Forkutsa I, Sharafutdinova N. Use of electrical conductivity instead of soluble salts for soil salinity monitoring in Central Asia. Irrigation and Drainage Systems. 2000; 14: 199–205.

[56] Daniells IG, Holland JF, Young RR, Alston CL, Bernardi AL. Relationship between yield of grain sorghum (Sorghum bicolor) and soil salinity under field conditions. Australian Journal of Experimental Agriculture. 2001; 41: 211–217.

[57] Mondal MK, Bhuiyan SI, Franco DT. Soil salinity reduction and prediction of salt dynamics in the coastal ricelands of Bangladesh. Agricultural Water Management. 2001; 47: 9–23.

[58] Dehaan RL, Taylor GR. Field-derived spectra of salinized soils and vegetation as indicators of irrigation-induced soil salinization. Remote Sensing of Environment. 2002; 80: 406–417.

[59] Dunn BW, Beecher HG, Batten GD, Ciavarella S. The potential of near-infrared reflectance spectroscopy for soil analysis—a case study from the Riverine Plain of south-eastern Australia. Australian Journal of Experimental Agriculture. 2002; 42: 607–614.

[60] Rogers ME. Irrigating perennial pasture with saline water: effects on soil chemistry, pasture production and composition. Australian Journal of Experimental Agriculture. 2002; 42: 265–272.

[61] Hernández Bastida JA, Vela N, Ortiz R. Electrolytic conductivity of semiarid soils (SE Spain) in relation to ion composition. Arid Land Research and Management. 2004; 18: 265–281.

[62] Piernik A. Vegetation-environment relations on inland saline habitats in Central Poland. Phytocoenologia. 2005; 35: 19–37.

[63] Barrett G. Vegetation communities on the shores of a salt lake in semi-arid Western Australia. Journal of Arid Environments. 2006; 67: 77–89.

[64] Morari F, Lugato E, Giardini L. Olsen phosphorus, exchangeable cations and salinity in two long-term experiments of north-eastern Italy and assessment of soil quality evolution. Agriculture, Ecosystems and Environment. 2008; 124: 85–96.

[65] Sigua G, Hudnall WH. Kriging analysis of soil properties. Journal of Soil and Sediments. 2008; 8: 193–202.

[66] Bennett SJ, Barrett-Lennard EG, Colmer TD. Salinity and waterlogging as constraints to saltland pasture production: A review. Agriculture Ecosystems & Environment. 2009; 129: 349–360.

[67] Moral FJ, Terrón JM, da Silva JRM. Delineation of management zones using mobile measurements of soil apparent electrical conductivity and multivariate geostatistical techniques. Soil & Tillage Research. 2010; 106: 335–343.

[68] Tóth T. Medium-term vegetation dynamics and their association with edaphic conditions in two Hungarian saline grassland communities. Grassland Science. 2010; 56: 13–18.

[69] Elgharably A. Wheat response to combined application of nitrogen and phosphorus in a saline sandy loam soil. Soil Science and Plant Nutrition. 2011; 57: 396–402.

[70] Yang F, Zhang G, Yin X, Liu Z. Field-scale spatial variation of saline-sodic soil and its relation with environmental factors in Western Songnen Plain of China. International Journal of Environmental Research and Public Health. 2011; 8: 374–387. doi: 10.3390/ijerph8020374 21556192 10.3390/ijerph8020374 PMC3084467

[71] García E, García C, Hernández T. Evaluation of the suitability of using large amounts of urban wastes for degraded arid soil restoration and C fixation. European Journal of Soil Science. 2012; 63: 650–658.

[72] König P. Plant life in the Umm as Samim, Oman—A case study in a major inland sabkha. Journal of Arid Environments. 2012; 85: 122–127.

[73] Harper RJ, Okom AEA, Stilwell AT, Tibbett M, Dean C, George SJ, et al Reforesting degraded agricultural landscapes with Eucalypts: Effects on carbon storage and soil fertility after 26 years. Agriculture Ecosystems & Environment. 2012; 163: 3–13.

[74] Karlen DL, Kovar JL, Cambardella CA, Colvin TS. Thirty-year tillage effects on crop yield and soil fertility indicators. Soil & Tillage Research. 2013; 130: 24–41.

[75] Klimanov AV, Vorob’eva LA, Novikova AF, Konyushkova MV. The nature of alkalinity in virgin and anthropogenically modified solonetzes of Northern Kalmykia. Eurasian Soil Science. 2014; 47: 266–275.

[76] Nikiforova EM, Kasimov NS, Kosheleva NE. Long-term dynamics of the anthropogenic salinization of soils in Moscow by the example of Eastern District. Eurasian Soil Science. 2014; 47: 203–215.

[77] Quintero JM, Enamorado S, Mas JL, Abril JM, Polvillo O, Delgado A. Phosphogypsum amendments and irrigation with acidulated water affect tomato nutrition in reclaimed marsh soils from SW Spain. Spanish Journal of Agricultural Research. 2014; 12: 809–819.

[78] Al-Busaidi A, Yamamoto T, Bahheit C, Cookson P. Soil salinity assessment by some destructive and non-destructive methods in calcareous soils. Journal of the Japanese Society of Soil Physics. 2006; 104: 27–40.

[79] Bilgili AV, van Es HM, Akbas F, Durak A, Hively WD. Visible-near infrared reflectance spectroscopy for assessment of soil properties in a semi-arid area of Turkey. Journal of Arid Environments. 2010; 74: 229–238.

[80] Nasiev BN, Eleshev R. Modern state of the soils of the flood irrigation systems in the Semidesert Zone. Eurasian Soil Science. 2014; 47: 613–620.

[81] Sidike A, Zhao S, Wen Y. Estimating soil salinity in Pingluo County of China using QuickBird data and soil reflectance spectra. International Journal of Applied Earth Observation and Geoinformation. 2014; 26: 156–175.

[82] Kok B, George PR, Strecht J. Saltland revegetation with salt-tolerant shrubs. Reclamation and Revegetation Research. 1986; 5: 501–507.

[83] McFadden LD, McDonald EV, Wells SG, Anderson K, Quade J, Forman SL. The vesicular layer and carbonate collars of desert soils and pavements: formation, age and relation to climate change. Geomorphology. 1998; 24: 101–145.

[84] Cemek B, Güler M, Kiliç K, Demir Y, Arslan H. Assessment of spatial variability in some soil properties as related to soil salinity and alkalinity in Bafra plain in northern Turkey. Monitoring and Assessment. 2007; 124: 223–234. 16957860 10.1007/s10661-006-9220-y

[85] Demir Y, Erşahin S, Güler M, Cemek B, Günal H, Arslan H. Spatial variability of depth and salinity of groundwater under irrigated Ustifluvents in the Middle Black Sea Region of Turkey. Environmental Monitoring and Assessment. 2009; 158: 279–294. doi: 10.1007/s10661-008-0582-1 19016338 10.1007/s10661-008-0582-1

[86] Mousavifard SE, Momtaz H, Sepehr E, Davatgar N, Sadaghiani MH. Determining and mapping some soil physico-chemical properties using geostatistical and GIS techniques in the Naqade region, Iran. Archives of Agronomy and Soil Science. 2013; 59: 1573–1589.

[87] Simón M, Cabezas O, García I Martínez P. A new method for the estimation of total dissolved salts in saturation extracts of soils from electrical conductivity. European Journal of Soil Science. 1994; 45: 153–157.

[88] Domínguez-Beisiegel M, Castañeda C, Herrero J. Two microenvironments at the soil surface of saline wetlands in Monegros, Spain. Soil Science Society of America Journal. 2013; 77: 653–663.

[89] He YB, DeSutter T, Prunty L, Hopkins D, Jia XH, Wysocki DA. Evaluation of 1:5 soil to water extract electrical conductivity methods. Geoderma. 2012; 185: 12–17.

[90] Sonneveld C, van den Ende J. Soil analysis by means of a 1:2 volume extract. Plant and Soil. 1971; 35: 505–516.

[91] Soil Survey Staff. Soil survey field and laboratory methods manual. Soil Survey Investigations Report 51, ver. 2.0. USDA-NRCS; 2014.

[92] Artieda O, Herrero J, Drohan PJ. A refinement of the differential water loss method for gypsum determination in soils. Soil Science Society of America Journal. 2006; 70: 1932–1935.

[93] Casby-Horton S, Herrero J, Rolong NA. Gypsum soils—Their morphology, classification, function, and landscapes. Advances in Agronomy. 2015; 130: 231–290.

[94] Herrero J, Artieda O, Hudnall WH. Gypsum, a tricky material. Soil Science Society of America Journal. 2009; 73: 1757–1763.

[95] Stiven GA, Khan MA. Saturation percentage as a measure of soil texture in the Lower Indus Basin. Journal of Soil Science. 1966; 17: 255–263.

[96] Juniper S, Abbott L. Vesicular-arbuscular mycorrhizas and soil salinity. Mycorrhiza. 1993; 4: 45–57. 10.1007/s00572-006-0046-9 16525784

[97] Peck AJ, Hatton T. Salinity and the discharge of salts from catchments in Australia. Journal of Hydrology. 2003; 272: 191–202.

[98] Rhoades JD, Chanduvi F, Lesch SM. Soil salinity assessment. Methods and interpretation of electrical conductivity measurements. FAO Irrigation and Drainage Paper 57. FAO, Roma; 1999.

[99] Herrero J, Bercero A. La salinidad en el nuevo regadío de Quinto (Zaragoza). Suelo y Planta. 1991; 1: 585–602.

[100] Huang J, Nhan T, Wong VNL, Johnston SG, Lark RM, Triantafilis J. Digital soil mapping of a coastal acid sulphate soil landscape. Soil Research. 2014; 52: 327–339.

[101] Ceuppens J, Wopereis MCS, Miézan KM. Soil salinization processes in rice irrigation schemes in the Senegal River Delta. Soil Science Society of America Journal. 1997; 61: 1122–1130.

[102] Ceuppens J, Wopereis MCS. Impact of non-drained irrigated rice cropping on soil salinization in the Senegal River Delta. Geoderma. 1999; 92: 125–140.

[103] Bennett DL, George RJ, Whitfield B. The use of ground EM systems to accurately assess salt store and help define land management options for salinity management. Exploratory Geophysics. 2000; 31: 249–254.

[104] Díaz L, Herrero J. Salinity estimates in irrigated soils using electromagnetic induction. Soil Science. 1992; 154: 151–157.

[105] Lesch SM, Herrero J, Rhoades JD. Monitoring for temporal changes in soil salinity using electromagnetic induction techniques. Soil Science Society of America Journal. 1998; 62: 232–242.

[106] Playán E, Pérez-Coveta O, Martínez-Cob A, Herrero J, García-Navarro P, Latorre B, et al Overland water and salt flows in a set of rice paddies. Agricultural Water Management. 2008; 95: 645–658.

[107] Herrero J, Hudnall WH. Measurement of soil salinity using electromagnetic induction in a paddy with a densic pan and shallow water table. Paddy and Water Environment. 2014; 12: 263–274.

[108] Shi Z, Li Y, Wang RC, Makeschine F. Assessment of temporal and spatial variability of soil salinity in a coastal saline field. Environmental Geology. 2005; 48: 171–178.

[109] Goff A, Huang J, Wong VNL, Monteiro Santos FA, Wege R Triantafilis J. Electromagnetic conductivity imaging of soil salinity in an estuarine–alluvial landscape. Soil Science Society of America Journal. 2014; 78: 1686–1693.

[110] Weindorf DC, Herrero J, Castañeda C, Bakr N, Swanhart S. Direct soil gypsum quantification via portable X-ray fluorescence spectrometry. Soil Science Society of America Journal. 2013; 77: 2071–2077.

[111] Swanhart S, Weindorf DC, Chakraborty S, Bakr N, Zhu Y, Nelson C, et al Soil salinity measurement via portable X-ray fluorescence spectrometry. Soil Science. 2015; 179: 417–423.

[112] Sumner ME, Rengasamy P, Naidu R. Sodic soils: a reappraisal In: Sumner ME, Naidu R, editors. Sodic soils: distribution, properties, management, and environmental consequences. Oxford University Press, New York; 1998 pp. 3–17.

