# Supplementary material for: Two Fixed Ratio Dilutions for Soil Salinity Monitoring in Hypersaline Wetlands
Source: PLoS One. 2015 May 22;10(5):e0126493. doi: 10.1371/journal.pone.0126493 (PMC4441515; doi:10.1371/journal.pone.0126493)
Supplement: S1 Table — (PDF) [file pone.0126493.s001.pdf]

S1 Table. UTM coordinates (European Datum ED 50) of the 59 sampling sites with their vegetation and the depth of the augerings.

Wetland	Site	Coord. X	Coord. Y	Vegetation	Depth, cm
**Amarga Alta**	AMA-1	745955.9	4587215.4	*Frankenia pulverulenta*	200
	AMA-2	746062.3	4587178.9	Bare soil	200
	AMA-3	746211.5	4587132.2	*Suaeda vera*	150
	AMA-4	746299.3	4587100.4	Winter cereal	150
	AMA-5	746426.0	4587058.4	*Salsolo-Artemisietum*	50
**Amarga Baja**	AMB-01	748475.2	4588286.4	*Aeluropus littoralis*	150
	AMB-02	748522.6	4588231.2	Bare soil	175
	AMB-03	748563.0	4588184.6	*Salicornia ramosissima*	200
	AMB-04	748617.4	4588120.1	*Lygeum spartum*	200
	AMB-05	748677.2	4588051.7	*Salsolo-Artemisietum*	150
	AMB-06	748789.9	4588106.9	Winter cereal	150
**Camarón**	CMR-01	726904.7	4587453.9	Bare soil	140
	CMR-02	727060.2	4587377.6		200
	CMR-03	727195.8	4587322.9		180
**Guallar**	GLR-01	731699.0	4587997.0	Bare soil	140
	GLR-02	731812.0	4587935.0		260
	GLR-03	732020.0	4587840.0		120
	GLR-04	732070.0	4587829.0	*S. vera*	200
	GLR-05	732101.0	4587816.0		180
	GLR-06	732149.0	4587722.0		60
	GLR-07	732089.0	4587742.0		160
	GLR-08	732102.0	4587705.0	Winter cereal	80
	GLR-09	732138.0	4587687.0		80
	GLR-10	732173.0	4587678.0		80
**Gramenosa**	GRM-01	742287.0	4586591.0	Winter cereal	100
	GRM-02	742318.0	4586636.0		100
	GRM-03	742338.0	4586669.0		100
	GRM-04	742350.0	4586689.0	*S. vera*	75
	GRM-05	742382.0	4586733.0	*S. vera* + Ruderal nitrophilous scrubs and grasses	150
	GRM-06	742412.0	4586754.0		150
	GRM-07	742443.0	4586793.0		150
	GRM-08	742479.0	4586837.0		150
	GRM-09	742507.0	4586878.0		150
	GRM-10	742528.0	4586925.0	*S. vera* + *F. pulverulenta*	150
	GRM-11	742551.0	4586937.0	Winter cereal	125
**Muerte**	MRT-01	728907.9	4587270.2	Bare soil	120
	MRT-02	728982.0	4587179.2		140
	MRT-03	729074.5	4587058.7		160
**Pez**	PEZ-01	729086.5	4584561.7	Bare soil	180
	PEZ-02	729147.5	4584590.3		180
	PEZ-03	729179.1	4584599.0		200
**Piñol**	PNL-01	729373.4	4587926.2	Bare soil	160
	PNL-02	729461.5	4587891.2		160
	PNL-03	729568.0	4587859.0		160
**Rebollón**	RBL-01	725492.5	4584433.4	Bare soil	100
	RBL-02	725580.6	4584398.3		60
	RBL-03	725641.8	4584370.2		80

…/…

S1 Table 1. (continued).

Wetland	Site	Coord. X	Coord. Y	Vegetation	Depth, cm
**Rollico**	RLL-01	726009.0	4585867.0	Bare soil	100
	RLL-03	726263.0	4585720.0		100
	RLL-04	726345.0	4585663.0	*Arthrocnemum macrostachyum*	120
	RLL-05	726384.0	4585639.0		120
	RLL-06	726418.0	4585611.0	*A. macrostachyum + S. vera*	80
	RLL-07	726437.0	4585607.0	Winter cereal	100
	RLL-08	726485.0	4585581.0		60
	RLL-09	726528.0	4585555.0		60
	RLL-10	726572.0	4585536.0		100
	RLL-11	726594.0	4585522.0		80
	RLL-12	726649.0	4585488.0		100
	RLL-13	726701.0	4585463.0		40
