# Supplementary material for: Two Fixed Ratio Dilutions for Soil Salinity Monitoring in Hypersaline Wetlands
Source: PLoS One. 2015 May 22;10(5):e0126493. doi: 10.1371/journal.pone.0126493 (PMC4441515; doi:10.1371/journal.pone.0126493)
Supplement: S2 Table — (PDF) [file pone.0126493.s002.pdf]

**S2 Table. Analytical data of the soil samples.**

| Soil sample | EC1:5 dS $m^{-1}$ | EC1:10 dS $m^{-1}$ | Cl1:10 meq $L^{-1}$ | ECe dS $m^{-1}$ | SP % | Gypsum % | CCE % |
|---|---|---|---|---|---|---|---|
| AMA-1/1 | 13.25 | 8.07 | | 76.10 | 54 | 60.0 | 16.7 |
| AMA-1/2 | 10.37 | 6.60 | | 56.60 | 58 | 81.2 | 7.1 |
| AMA-1/3 | 11.68 | 7.21 | | 62.40 | 59 | 78.8 | 8.2 |
| AMA-1/4 | 14.89 | 8.70 | | 85.00 | 52 | 70.8 | 12.9 |
| AMA-1/5 | 12.97 | 7.87 | | 81.50 | 50 | 74.4 | 11.2 |
| AMA-1/6 | 13.05 | 7.89 | | 82.30 | 48 | 75.0 | 8.1 |
| AMA-1/7 | 11.13 | 6.99 | | 88.80 | 37 | 87.1 | 5.1 |
| AMA-1/8 | 13.25 | 8.07 | | 99.30 | 37 | 75.6 | 9.7 |
| AMA-2/1 | 13.35 | 8.38 | | 68.10 | 67 | 58.2 | 17.0 |
| AMA-2/2 | 9.96 | 6.31 | | 49.50 | 63 | 78.8 | 8.7 |
| AMA-2/3 | 9.76 | 6.10 | | 67.70 | 44 | 89.4 | 3.3 |
| AMA-2/4 | 9.48 | 6.03 | | 70.00 | 41 | 91.3 | 2.1 |
| AMA-2/5 | 10.94 | 7.05 | | 80.20 | 41 | 88.3 | 2.0 |
| AMA-2/6 | 10.10 | 6.47 | | 77.30 | 42 | 85.2 | 2.3 |
| AMA-2/7 | 10.64 | 6.78 | | 78.00 | 39 | 81.1 | 2.2 |
| AMA-2/8 | 10.28 | 6.54 | | 86.20 | 32 | 84.5 | 2.7 |
| AMA-3/1 | 10.57 | 6.73 | | 74.50 | 40 | 66.8 | 11.9 |
| AMA-3/2 | 10.46 | 6.66 | | 82.40 | 38 | 89.6 | 3.6 |
| AMA-3/3 | 10.26 | 6.36 | | 86.50 | 36 | 93.4 | 2.2 |
| AMA-3/4 | 10.43 | 6.60 | | 92.80 | 24 | 76.0 | 14.0 |
| AMA-3/5 | 6.09 | 4.28 | | 64.30 | 30 | 65.9 | 29.9 |
| AMA-3/6 | 8.74 | 5.66 | | 84.80 | 28 | 69.4 | 16.3 |
| AMA-4/1 | 2.51 | 2.36 | | 4.52 | 41 | 48.1 | 19.5 |
| AMA-4/2 | 3.12 | 2.78 | | 8.37 | 45 | 44.5 | 21.9 |
| AMA-4/3 | 4.00 | 3.13 | | 13.60 | 47 | 26.7 | 35.5 |
| AMA-4/4 | 3.88 | 3.07 | | 13.39 | 52 | 25.6 | 41.5 |
| AMA-4/5 | 3.60 | 2.91 | | 13.80 | 42 | 70.4 | 19.1 |
| AMA-4/6 | 3.04 | 2.62 | | 10.52 | 36 | 77.2 | 11.5 |
| AMA-5/1 | 2.56 | 2.01 | | 5.41 | 50 | 6.6 | 38.4 |
| AMA-5/2 | 4.17 | 2.82 | | 17.39 | 52 | 7.9 | 45.8 |
| AMB-1/1 | 11.77 | 7.24 | | 49.10 | 44 | 60.3 | 18.4 |
| AMB-1/2 | 6.60 | 4.50 | | 74.70 | 48 | 84.1 | 6.2 |
| AMB-1/3 | 6.27 | 4.32 | | 43.90 | 36 | 85.8 | 4.3 |
| AMB-1/4 | 6.34 | 4.34 | | 44.60 | 31 | 89.5 | 2.7 |
| AMB-1/5 | 5.94 | 4.08 | | 40.80 | 35 | 55.7 | 15.4 |
| AMB-1/6 | 5.63 | 3.72 | | 29.23 | 47 | 30.3 | 22.6 |
| AMB-02/1 | 9.37 | 6.02 | | 60.30 | 50 | 76.5 | 5.9 |
| AMB-02/2 | 7.73 | 5.14 | | 63.00 | 41 | 86.4 | 2.3 |
| AMB-02/3 | 7.04 | 4.62 | | 55.30 | 37 | 89.4 | 0.5 |
| AMB-02/4 | 8.47 | 5.49 | | 65.00 | 35 | 83.8 | 1.1 |
| AMB-02/5 | 8.88 | 5.67 | | 68.20 | 35 | 81.0 | 3.0 |
| AMB-02/6 | 8.99 | 5.71 | | 33.90 | 36 | 61.6 | 18.6 |

| Soil sample | EC1:5 dS m$^{-1}$ | EC1:10 dS m$^{-1}$ | Cl1:10 meq L$^{-1}$ | ECe dS m$^{-1}$ | SP % | Gypsum % | CCE % |
|---|---|---|---|---|---|---|---|
| AMB-02/7 | 7.04 | 4.46 | | 53.10 | 34 | 34.7 | 40.5 |
| AMB-3/1 | 10.90 | 6.89 | | 83.30 | 39 | 82.9 | 4.2 |
| AMB-3/2 | 10.57 | 6.59 | | 90.40 | 31 | 86.6 | 1.0 |
| AMB-3/3 | 10.62 | 6.45 | | 94.00 | 29 | 86.4 | 1.6 |
| AMB-3/4 | 10.86 | 6.73 | | 95.40 | 31 | 83.9 | 1.5 |
| AMB-3/5 | 9.29 | 5.82 | | 82.90 | 32 | 84.1 | 1.5 |
| AMB-3/6 | 9.68 | 6.13 | | 92.20 | 28 | 63.0 | 17.3 |
| AMB-3/7 | 10.07 | 6.20 | | 74.60 | 41 | 21.0 | 46.3 |
| AMB-3/8 | 9.35 | 5.38 | | 58.40 | 52 | 9.7 | 51.9 |
| AMB-4/1 | 7.23 | 5.01 | | 41.70 | 48 | 35.9 | 26.4 |
| AMB-4/2 | 7.67 | 5.25 | | 53.60 | 39 | 54.5 | 17.7 |
| AMB-4/3 | 6.04 | 4.32 | | 42.60 | 37 | 80.4 | 7.9 |
| AMB-4/4 | 5.54 | 4.01 | | 37.20 | 34 | 85.3 | 6.3 |
| AMB-4/5 | 5.16 | 3.82 | | 36.00 | 33 | 84.5 | 5.5 |
| AMB-4/6 | 5.15 | 3.71 | | 37.80 | 31 | 87.1 | 4.7 |
| AMB-4/7 | 4.94 | 3.63 | | 32.80 | 33 | 83.8 | 5.3 |
| AMB-4/8 | 4.16 | 3.19 | | 25.30 | 34 | 88.2 | 3.8 |
| AMB-5/1 | 2.28 | 2.18 | | 3.53 | 29 | 7.9 | 48.3 |
| AMB-5/2 | 2.96 | 2.55 | | 7.66 | 33 | 8.4 | 46.3 |
| AMB-5/3 | 3.50 | 2.96 | | 11.10 | 38 | 31.4 | 38.8 |
| AMB-5/4 | 3.02 | 2.67 | | 8.93 | 45 | 56.7 | 29.2 |
| AMB-5/5 | 2.80 | 2.57 | | 7.70 | 40 | 36.1 | 43.6 |
| AMB-5/6 | 2.73 | 2.53 | | 7.35 | 42 | 19.8 | 57.7 |
| AMB-6/1 | 2.41 | 2.30 | | 4.24 | 34 | 77.9 | 7.7 |
| AMB-6/2 | 2.49 | 2.35 | | 5.13 | 31 | 85.4 | 4.4 |
| AMB-6/3 | 2.53 | 2.37 | | 6.10 | 30 | 88.2 | 5.8 |
| AMB-6/4 | 2.53 | 2.36 | | 5.97 | 30 | 88.6 | 5.7 |
| AMB-6/5 | 2.41 | 2.30 | | 4.97 | 30 | 94.2 | 3.8 |
| AMB-6/6 | 5.43 | 3.95 | | 45.20 | 30 | 69.9 | 22.7 |
| CMR-01/01 | 24.20 | 14.01 | 111.30 | 132.00 | 57 | 28.9 | 14.6 |
| CMR-01/02 | 21.10 | 13.04 | 104.80 | 111.10 | 62 | 26.9 | 14.0 |
| CMR-01/03 | 16.10 | 9.86 | 69.10 | 111.40 | 45 | 46.4 | 14.0 |
| CMR-01/04 | 15.50 | 9.20 | 64.10 | 124.50 | 32 | 53.4 | 12.8 |
| CMR-01/05 | 13.20 | 8.01 | 53.80 | 124.50 | 28 | 41.5 | 34.3 |
| CMR-01/06 | 14.00 | 8.31 | 57.00 | 137.00 | 25 | 51.5 | 31.9 |
| CMR-01/07 | 16.70 | 9.94 | 70.70 | 162.00 | 23 | | |
| CMR-02/01 | 22.00 | 12.69 | 97.40 | 126.90 | 51 | 38.0 | 14.2 |
| CMR-02/02 | 18.90 | 11.16 | 79.50 | 109.10 | 55 | 37.9 | 11.9 |
| CMR-02/03 | 17.80 | 10.49 | 74.50 | 105.20 | 48 | 47.3 | 8.6 |
| CMR-02/04 | 16.20 | 9.58 | 67.30 | 183.00 | 36 | 58.7 | 5.6 |
| CMR-02/05 | 16.70 | 10.23 | 73.60 | 122.80 | 40 | 59.1 | 6.0 |
| CMR-02/06 | 18.20 | 10.80 | 77.60 | 130.00 | 41 | 56.6 | 6.5 |
| CMR-02/07 | 17.80 | 10.29 | 74.60 | 127.90 | 41 | 44.8 | 13.0 |
| CMR-02/08 | 19.10 | 10.31 | 73.00 | 115.70 | 47 | 30.3 | 21.4 |

| Soil sample | EC1:5 dS m$^{-1}$ | EC1:10 dS m$^{-1}$ | Cl1:10 meq L$^{-1}$ | ECe dS m$^{-1}$ | SP % | Gypsum % | CCE % |
|---|---|---|---|---|---|---|---|
| CMR-02/09 | 18.60 | 11.15 | 83.00 | 94.80 | 47 | 40.9 | 18.7 |
| CMR-02/10 | 14.60 | 8.73 | 61.60 | 101.60 | 43 | 46.8 | 15.3 |
| CMR-03/01 | 23.60 | 14.00 | 106.90 | 144.00 | 46 | 38.8 | 13.3 |
| CMR-03/02 | 20.20 | 11.82 | 86.20 | 123.40 | 48 | 44.1 | 10.6 |
| CMR-03/03 | 17.00 | 9.81 | 68.90 | 109.80 | 45 | 52.5 | 8.2 |
| CMR-03/04 | 13.70 | 8.31 | 54.90 | 125.30 | 29 | 71.1 | 3.5 |
| CMR-03/05 | 15.20 | 9.09 | 61.70 | 119.00 | 36 | 63.7 | 4.1 |
| CMR-03/06 | 15.70 | 9.42 | 66.00 | 120.30 | 38 | 60.9 | 4.5 |
| CMR-03/07 | 13.20 | 8.40 | 55.90 | 109.90 | 35 | 54.1 | 9.4 |
| CMR-03/08 | 14.80 | 9.02 | 60.00 | 87.40 | 46 | 34.4 | 20.6 |
| CMR-03/09 | 15.70 | 9.22 | 62.90 | 106.20 | 44 | 37.7 | 19.9 |
| GLR-01/01 | 28.40 | 16.20 | 126.90 | 139.00 | 56 | 48.8 | 16.7 |
| GLR-01/02 | 23.70 | 14.20 | 109.30 | 138.00 | 55 | 42.9 | 19.3 |
| GLR-01/03 | 14.60 | 9.07 | 59.50 | 102.00 | 40 | 48.1 | 16.9 |
| GLR-01/04 | 14.00 | 8.71 | 55.00 | 100.40 | 39 | 57.4 | 10.1 |
| GLR-01/05 | 10.00 | 6.24 | 33.50 | 93.80 | 27 | 65.9 | 8.6 |
| GLR-01/06 | 9.74 | 6.08 | 32.50 | 96.80 | 30 | 60.5 | 26.8 |
| GLR-01/07 | 9.58 | 6.28 | 36.80 | 98.00 | 25 | 15.2 | 72.6 |
| GLR-02/01 | 21.50 | 13.10 | 95.80 | 121.30 | 52 | 59.8 | 10.9 |
| GLR-02/02 | 22.30 | 12.50 | 91.60 | 117.00 | 49 | 60.9 | 7.9 |
| GLR-02/03 | 15.90 | 9.12 | 60.90 | 127.40 | 32 | 75.8 | 3.5 |
| GLR-02/04 | 13.30 | 8.11 | 50.20 | 114.20 | 30 | 75.7 | 2.5 |
| GLR-02/05 | 14.30 | 8.62 | 58.02 | 112.50 | 33 | 76.9 | 2.2 |
| GLR-02/06 | 13.80 | 8.35 | 51.10 | 109.40 | 32 | 78.2 | 1.9 |
| GLR-02/07 | 17.50 | 9.80 | 65.50 | 125.70 | 38 | 66.7 | 3.0 |
| GLR-02/08 | 15.60 | 9.25 | 61.70 | 123.50 | 32 | 76.3 | 1.9 |
| GLR-02/09 | 14.30 | 8.58 | 57.27 | 125.50 | 25 | 76.7 | 2.3 |
| GLR-02/10 | 12.58 | 8.20 | 52.40 | 119.60 | 27 | 80.0 | 4.7 |
| GLR-02/11 | 12.66 | 7.37 | 44.30 | 106.20 | 33 | 73.2 | 10.4 |
| GLR-02/12 | 11.76 | 7.17 | 41.00 | 123.40 | 29 | 70.2 | 13.0 |
| GLR-02/13 | 13.70 | 8.18 | 52.10 | 109.70 | 33 | 17.4 | 57.4 |
| GLR-03/01 | 18.30 | 9.91 | 67.20 | 138.00 | 31 | 69.9 | 9.9 |
| GLR-03/02 | 16.90 | 10.22 | 68.70 | 132.00 | 33 | 66.3 | 9.5 |
| GLR-03/03 | 12.60 | 8.28 | 51.80 | 118.60 | 26 | 73.5 | 6.0 |
| GLR-03/04 | 11.04 | 7.13 | 40.80 | 111.20 | 23 | 76.4 | 3.2 |
| GLR-03/05 | 10.18 | 6.49 | 36.00 | 121.00 | 21 | 77.6 | 0.7 |
| GLR-03/06 | 10.69 | 6.45 | 37.00 | 108.90 | 21 | 71.3 | 2.1 |
| GLR-04/01 | 8.63 | 5.66 | 28.80 | 73.30 | 31 | 73.6 | 12.4 |
| GLR-04/02 | 9.12 | 5.51 | 27.40 | 74.70 | 30 | 76.6 | 10.2 |
| GLR-04/03 | 7.65 | 5.01 | 22.30 | 60.60 | 34 | 78.1 | 8.1 |
| GLR-04/04 | 6.11 | 4.26 | 18.00 | 49.20 | 31 | 90.3 | 4.1 |
| GLR-04/05 | 7.12 | 4.62 | 21.00 | 62.80 | 30 | 62.8 | 7.7 |
| GLR-04/06 | 7.78 | 5.18 | 23.70 | 68.20 | 29 | 82.6 | 7.0 |
| GLR-04/07 | 8.19 | 5.52 | 27.30 | 78.20 | 28 | 86.5 | 4.8 |

| Soil sample | EC1:5<br>dS m$^{-1}$ | EC1:10<br>dS m$^{-1}$ | Cl1:10<br>meq L$^{-1}$ | ECe<br>dS m$^{-1}$ | SP<br>% | Gypsum<br>% | CCE<br>% |
|---|---|---|---|---|---|---|---|
| GLR-04/08 | 8.64 | 5.50 | 26.80 | 74.80 | 29 | 76.5 | 12.5 |
| GLR-04/09 | 7.56 | 5.00 | 22.80 | 72.80 | 25 | 51.6 | 32.0 |
| GLR-04/10 | 7.89 | 5.17 | 25.40 | 78.90 | 24 | 29.6 | 60.4 |
| GLR-05/1 | 10.84 | 6.57 | 35.80 | 60.50 | 54 | 8.4 | 34.3 |
| GLR-05/2 | 10.02 | 6.32 | 29.70 | 62.30 | 44 | 20.0 | 33.0 |
| GLR-05/3 | 9.23 | 5.94 | 28.80 | 60.70 | 42 | 34.7 | 25.5 |
| GLR-05/4 | 7.52 | 4.93 | 20.30 | 58.80 | 34 | 58.5 | 15.1 |
| GLR-05/5 | 8.01 | 5.12 | 23.20 | 68.00 | 34 | 68.7 | 10.9 |
| GLR-05/6 | 7.80 | 4.92 | 21.50 | 67.60 | 29 | 80.2 | 7.8 |
| GLR-05/7 | 6.54 | 3.97 | 13.70 | 66.30 | 25 | 63.6 | 28.5 |
| GLR-05/8 | 6.35 | 4.28 | 16.60 | 73.90 | 24 | 56.6 | 37.8 |
| GLR-05/9 | 5.69 | 3.88 | 13.80 | 61.60 | 23 | 75.1 | 22.2 |
| GLR-06/1 | 3.90 | 3.03 | 7.20 | 16.00 | 44 | 10.7 | 44.1 |
| GLR-06/2 | 4.54 | 3.42 | 8.70 | 21.40 | 45 | 13.1 | 47.5 |
| GLR-06/3 | 4.45 | 3.38 | 8.90 | 21.50 | 41 | 17.3 | 55.5 |
| GLR-07/1 | 6.02 | 4.17 | 14.10 | 33.20 | 44 | 34.1 | 30.3 |
| GLR-07/2 | 7.90 | 5.18 | 19.90 | 50.80 | 41 | 32.2 | 33.8 |
| GLR-07/3 | 7.50 | 5.00 | 20.20 | 58.30 | 32 | 57.4 | 22.6 |
| GLR-07/4 | 7.50 | 5.24 | 22.50 | 61.80 | 32 | 47.2 | 28.7 |
| GLR-07/5 | 8.19 | 5.09 | 21.70 | 54.10 | 37 | 28.9 | 50.9 |
| GLR-07/6 | 8.54 | 5.40 | 25.90 | 54.60 | 43 | 43.0 | 33.2 |
| GLR-07/7 | 4.37 | 3.34 | 10.00 | 39.60 | 24 | 91.3 | 5.5 |
| GLR-07/8 | 4.36 | 3.27 | 10.80 | 33.20 | 26 | 96.4 | 3.1 |
| GLR-08/1 | 3.47 | 2.76 | 5.20 | 12.00 | 45 | 9.8 | 50.1 |
| GLR-08/2 | 4.36 | 3.30 | 7.80 | 18.40 | 45 | 36.8 | 41.2 |
| GLR-08/3 | 4.35 | 3.31 | 7.00 | 21.00 | 42 | 43.9 | 39.1 |
| GLR-08/4 | 3.93 | 3.06 | 6.20 | 18.80 | 37 | 8.8 | 47.1 |
| GLR-09/1 | 2.88 | 2.47 | 3.50 | 7.61 | 43 | 5.9 | 53.0 |
| GLR-09/2 | 3.37 | 2.65 | 3.10 | 11.71 | 43 | 5.7 | 56.1 |
| GLR-09/3 | 3.60 | 2.86 | 3.90 | 13.60 | 43 | 7.0 | 53.9 |
| GLR-09/4 | 3.53 | 2.87 | 3.80 | 13.40 | 42 | 20.0 | 46.9 |
| GLR-10/01 | 1.69 | 0.97 | 2.60 | 6.21 | 44 | 2.9 | 55.9 |
| GLR-10/02 | 3.10 | 2.38 | 2.30 | 9.17 | 44 | 4.6 | 56.6 |
| GLR-10/03 | 3.10 | 2.64 | 2.90 | 10.73 | 38 | 9.3 | 67.1 |
| GLR-10/04 | 2.77 | 2.49 | 2.00 | 8.60 | 30 | 75.5 | 18.7 |
| GRM-01/1 | 0.41 | 0.33 | 2.20 | 2.32 | 38 | 1.9 | 51.2 |
| GRM-01/2 | 0.86 | 0.58 | 1.90 | 2.98 | 44 | 2.1 | 51.8 |
| GRM-01/3 | 1.00 | 0.64 | 2.90 | 4.04 | 47 | 1.9 | 57.4 |
| GRM-01/4 | 2.66 | 2.39 | 1.10 | 5.19 | 36 | 39.2 | 37.9 |
| GRM-02/1 | 1.40 | 0.80 | 1.00 | 3.08 | 41 | 2.6 | 52.6 |
| GRM-02/2 | 2.03 | 1.49 | 0.90 | 4.62 | 44 | 3.5 | 51.0 |
| GRM-02/3 | 2.55 | 1.47 | 0.50 | 6.06 | 45 | 2.6 | 59.9 |
| GRM-02/4 | 2.89 | 2.31 | 0.60 | 6.32 | 43 | 4.3 | 62.3 |
| GRM-03/1 | 3.03 | 1.93 | 2.90 | 8.63 | 45 | 3.3 | 57.6 |

| Soil sample | EC1:5 dS m$^{-1}$ | EC1:10 dS m$^{-1}$ | Cl1:10 meq L$^{-1}$ | ECe dS m$^{-1}$ | SP % | Gypsum % | CCE % |
|---|---|---|---|---|---|---|---|
| GRM-03/2 | 3.54 | 2.85 | 4.10 | 12.80 | 43 | 6.5 | 57.5 |
| GRM-03/3 | 3.53 | 2.91 | 6.10 | 17.00 | 33 | 58.4 | 24.5 |
| GRM-03/4 | 3.89 | 3.05 | 6.70 | 24.00 | 28 | 56.0 | 28.1 |
| GRM-04/1 | 8.51 | 5.48 | 24.30 | 54.60 | 39 | 12.7 | 41.0 |
| GRM-04/2 | 7.75 | 4.98 | 20.70 | 51.70 | 37 | 11.7 | 49.4 |
| GRM-04/3 | 5.86 | 4.53 | 17.60 | 50.70 | 29 | 47.2 | 30.1 |
| GRM-05/1 | 4.06 | 3.02 | 5.90 | 14.30 | 49 | 8.6 | 39.6 |
| GRM-05/2 | 5.30 | 3.78 | 10.40 | 25.10 | 45 | 15.0 | 40.4 |
| GRM-05/3 | 4.51 | 3.38 | 8.70 | 23.80 | 37 | 65.7 | 15.8 |
| GRM-05/4 | 5.11 | 3.68 | 10.20 | 26.10 | 43 | 58.4 | 17.1 |
| GRM-05/5 | 5.33 | 3.74 | 11.30 | 30.80 | 39 | 64.6 | 12.3 |
| GRM-05/6 | 5.71 | 4.00 | 12.50 | 32.90 | 40 | 58.5 | 15.9 |
| GRM-06/1 | 6.50 | 4.35 | 15.90 | 32.40 | 49 | 13.1 | 35.6 |
| GRM-06/2 | 9.11 | 5.80 | 28.00 | 51.20 | 46 | 25.8 | 33.5 |
| GRM-06/3 | 6.96 | 5.03 | 20.90 | 51.70 | 34 | 68.5 | 13.1 |
| GRM-06/4 | 8.25 | 5.51 | 25.10 | 53.90 | 42 | 57.7 | 16.0 |
| GRM-06/5 | 7.85 | 4.91 | 20.50 | 60.30 | 33 | 68.5 | 9.6 |
| GRM-06/6 | 8.61 | 5.46 | 23.70 | 62.20 | 38 | 60.2 | 17.8 |
| GRM-07/1 | 3.65 | 2.79 | 3.50 | 10.84 | 48 | 9.6 | 37.5 |
| GRM-07/2 | 6.88 | 4.61 | 19.70 | 40.90 | 41 | 52.0 | 22.6 |
| GRM-07/3 | 6.96 | 4.65 | 20.30 | 49.50 | 34 | 74.2 | 9.4 |
| GRM-07/4 | 6.50 | 4.36 | 17.30 | 52.70 | 29 | 76.6 | 5.3 |
| GRM-07/5 | 6.78 | 4.61 | 18.60 | 46.60 | 36 | 66.2 | 9.9 |
| GRM-07/6 | 7.41 | 5.16 | 21.40 | 49.90 | 37 | 58.1 | 15.4 |
| GRM-08/1 | 5.37 | 3.87 | 12.20 | 24.00 | 48 | 10.5 | 34.9 |
| GRM-08/2 | 8.49 | 5.65 | 27.90 | 54.70 | 41 | 75.3 | 21.9 |
| GRM-08/3 | 8.35 | 5.74 | 28.20 | 67.80 | 35 | 75.5 | 8.4 |
| GRM-08/4 | 7.48 | 5.22 | 24.30 | 60.90 | 31 | 75.4 | 6.9 |
| GRM-08/5 | 7.91 | 5.33 | 24.50 | 66.70 | 30 | 76.4 | 5.9 |
| GRM-08/6 | 9.64 | 6.73 | 33.40 | 57.80 | 43 | 42.6 | 21.7 |
| GRM-09/1 | 4.07 | 3.08 | 6.40 | 14.60 | 51 | 10.4 | 35.8 |
| GRM-09/2 | 8.90 | 5.70 | 27.40 | 53.00 | 46 | 30.2 | 32.9 |
| GRM-09/3 | 8.02 | 5.18 | 22.80 | 64.70 | 35 | 73.6 | 9.7 |
| GRM-09/4 | 8.62 | 5.62 | 26.80 | 66.60 | 35 | 68.7 | 10.5 |
| GRM-09/5 | 7.91 | 4.97 | 21.80 | 71.50 | 28 | 76.8 | 6.5 |
| GRM-09/6 | 8.98 | 5.75 | 27.80 | 64.90 | 35 | 63.1 | 12.6 |
| GRM-10/1 | 4.82 | 3.42 | 9.10 | 27.20 | 38 | 22.3 | 41.6 |
| GRM-10/2 | 5.88 | 4.12 | 15.20 | 49.10 | 25 | 60.0 | 27.0 |
| GRM-10/3 | 5.69 | 3.91 | 14.00 | 50.70 | 25 | 35.7 | 46.6 |
| GRM-10/4 | 4.35 | 3.36 | 10.00 | 56.80 | 24 | 36.2 | 50.5 |
| GRM-10/5 | 6.30 | 4.09 | 14.70 | 48.20 | 29 | 63.0 | 23.6 |
| GRM-10/6 | 6.71 | 4.50 | 17.00 | 29.90 | 61 | 20.2 | 38.8 |
| GRM-11/1 | 5.43 | 3.75 | 13.40 | 27.40 | 46 | 7.0 | 51.7 |
| GRM-11/2 | 6.11 | 4.30 | 16.70 | 33.70 | 45 | 28.9 | 40.7 |

| Soil sample | EC1:5 dS m$^{-1}$ | EC1:10 dS m$^{-1}$ | Cl1:10 meq L$^{-1}$ | ECe dS m$^{-1}$ | SP % | Gypsum % | CCE % |
|---|---|---|---|---|---|---|---|
| GRM-11/3 | 5.51 | 3.95 | 14.30 | 34.10 | 39 | 62.1 | 27.5 |
| GRM-11/4 | 5.33 | 3.84 | 13.70 | 38.70 | 30 | 52.1 | 25.4 |
| GRM-11/5 | 5.02 | 3.74 | 13.00 | 37.20 | 29 | 56.5 | 34.9 |
| MRT-01/1 | 20.60 | 12.73 | 94.70 | 147.00 | 36 | 58.4 | 14.0 |
| MRT-01/2 | 23.00 | 14.00 | 107.10 | 154.00 | 36 | 58.1 | 13.0 |
| MRT-01/3 | 19.60 | 12.59 | 94.80 | 125.70 | 40 | 38.2 | 23.4 |
| MRT-01/4 | 15.90 | 9.61 | 64.00 | 114.90 | 39 | 41.3 | 23.0 |
| MRT-01/5 | 23.80 | 12.99 | 97.80 | 144.00 | 43 | 21.2 | 40.6 |
| MRT-01/6 | 25.00 | 13.20 | 103.80 | 158.00 | 38 | 15.0 | 53.0 |
| MRT-02/1 | 25.50 | 14.90 | 112.70 | 136.00 | 48 | 59.7 | 11.5 |
| MRT-02/2 | 22.90 | 13.60 | 107.80 | 152.00 | 39 | 61.9 | 11.0 |
| MRT-02/3 | 22.30 | 12.82 | 98.10 | 135.00 | 43 | 51.8 | 14.4 |
| MRT-02/4 | 17.40 | 10.46 | 72.20 | 133.00 | 34 | 51.2 | 18.1 |
| MRT-02/5 | 16.80 | 10.18 | 70.50 | 139.00 | 32 | 48.1 | 20.4 |
| MRT-02/6 | 17.00 | 10.02 | 69.50 | 127.10 | 35 | 52.9 | 18.4 |
| MRT-02/7 | 18.80 | 11.04 | 79.50 | 146.00 | 29 | 39.0 | 35.5 |
| MRT-03/1 | 20.50 | 12.42 | 93.10 | 163.00 | 27 | 79.0 | 7.3 |
| MRT-03/2 | 18.40 | 10.89 | 79.10 | 144.00 | 31 | 73.3 | 8.2 |
| MRT-03/3 | 17.40 | 10.42 | 71.80 | 126.70 | 35 | 74.3 | 7.3 |
| MRT-03/4 | 18.30 | 10.00 | 67.70 | 131.00 | 33 | 64.8 | 11.9 |
| MRT-03/5 | 16.80 | 9.84 | 65.20 | 132.00 | 32 | 51.9 | 17.1 |
| MRT-03/6 | 17.20 | 10.25 | 71.40 | 135.00 | 33 | 54.7 | 17.0 |
| MRT-03/7 | 19.20 | 11.05 | 78.80 | 129.70 | 39 | 45.7 | 20.4 |
| MRT-03/8 | 19.00 | 11.25 | 80.50 | 122.40 | 43 | 46.8 | 19.8 |
| PEZ-01/1 | 18.70 | 10.96 | 80.50 | 119.00 | 44 | 28.9 | 29.0 |
| PEZ-01/2 | 15.10 | 9.05 | 62.70 | 96.70 | 49 | 33.2 | 24.3 |
| PEZ-01/3 | 16.90 | 9.69 | 70.60 | 106.70 | 49 | 38.0 | 24.8 |
| PEZ-01/4 | 16.30 | 9.43 | 67.30 | 96.10 | 49 | 34.4 | 29.1 |
| PEZ-01/5 | 15.30 | 9.30 | 65.40 | 92.60 | 50 | 33.9 | 26.9 |
| PEZ-01/6 | 19.30 | 11.53 | 89.10 | 82.00 | 49 | 35.3 | 27.9 |
| PEZ-01/7 | 12.74 | 7.81 | 51.10 | 82.00 | 47 | 36.6 | 25.6 |
| PEZ-01/8 | 12.80 | 7.95 | 51.25 | 138.00 | 42 | 36.9 | 26.8 |
| PEZ-01/9 | 18.30 | 10.97 | 82.90 | 129.70 | 40 | | |
| PEZ-02/1 | 16.90 | 10.33 | 74.90 | 106.70 | 47 | 35.5 | 22.6 |
| PEZ-02/2 | 15.10 | 9.43 | 66.50 | 104.30 | 42 | 49.6 | 16.6 |
| PEZ-02/3 | 11.88 | 7.36 | 45.70 | 92.40 | 44 | 46.3 | 22.2 |
| PEZ-02/4 | 14.50 | 7.91 | 50.60 | 82.70 | 45 | 49.1 | 19.0 |
| PEZ-02/5 | 12.72 | 7.83 | 50.60 | 92.50 | 41 | 53.3 | 18.0 |
| PEZ-02/6 | 12.06 | 7.39 | 47.10 | 95.30 | 37 | 52.5 | 18.2 |
| PEZ-02/7 | 13.60 | 8.18 | 55.50 | 121.70 | 28 | 63.5 | 9.9 |
| PEZ-02/8 | 11.50 | 7.39 | 47.20 | 95.00 | 34 | 46.9 | 21.9 |
| PEZ-02/9 | 14.20 | 8.86 | 61.80 | 112.30 | 37 | 27.2 | 33.0 |
| PEZ-03/01 | 15.00 | 9.19 | 66.40 | 100.00 | 43 | 39.6 | 24.2 |
| PEZ-03/02 | 16.30 | 10.29 | 74.00 | 115.20 | 40 | 46.5 | 19.6 |

| Soil sample | EC1:5<br>dS $m^{-1}$ | EC1:10<br>dS $m^{-1}$ | Cl1:10<br>meq $L^{-1}$ | ECe<br>dS $m^{-1}$ | SP<br>% | Gypsum<br>% | CCE<br>% |
|---|---|---|---|---|---|---|---|
| PEZ-03/03 | 13.40 | 7.25 | 46.20 | 101.40 | 38 | 52.9 | 19.4 |
| PEZ-03/04 | 13.00 | 7.67 | 49.80 | 93.30 | 40 | 48.2 | 21.6 |
| PEZ-03/05 | 13.10 | 8.47 | 56.50 | 100.60 | 36 | 52.3 | 19.1 |
| PEZ-03/06 | 13.40 | 8.40 | 56.60 | 108.80 | 35 | 48.4 | 21.6 |
| PEZ-03/07 | 15.50 | 9.25 | 62.80 | 112.60 | 36 | 44.7 | 22.5 |
| PEZ-03/08 | 14.00 | 7.73 | 49.70 | 107.70 | 33 | 40.9 | 27.4 |
| PEZ-03/09 | 13.00 | 8.07 | 52.80 | 106.60 | 34 | 28.2 | 34.5 |
| PEZ-03/10 | 16.40 | 9.52 | 67.20 | 122.50 | 36 | 22.9 | 41.0 |
| PNL-01/1 | 18.40 | 10.52 | 70.70 | 113.10 | 57 | 36.6 | 14.1 |
| PNL-01/2 | 16.90 | 10.37 | 67.50 | 110.20 | 57 | 29.6 | 16.2 |
| PNL-01/3 | 24.90 | 14.00 | 106.90 | 122.30 | 61 | 36.0 | 14.5 |
| PNL-01/4 | 28.20 | 16.30 | 131.70 | 90.20 | 68 | 42.4 | 12.9 |
| PNL-01/5 | 27.70 | 15.40 | 124.30 | 133.00 | 60 | 37.6 | 15.9 |
| PNL-01/6 | 20.30 | 11.34 | 83.50 | 104.40 | 50 | 50.7 | 17.6 |
| PNL-01/7 | 10.75 | 6.87 | 41.90 | 96.60 | 40 | 18.2 | 49.7 |
| PNL-01/8 | 18.00 | 11.02 | 85.10 | 134.00 | 40 | 6.6 | 58.6 |
| PNL-02/1 | 17.60 | 10.52 | 70.90 | 121.80 | 53 | 40.1 | 14.0 |
| PNL-02/2 | 16.90 | 10.19 | 67.50 | 93.90 | 57 | 33.5 | 15.4 |
| PNL-02/3 | 15.20 | 10.50 | 71.10 | 81.60 | 64 | 30.0 | 15.9 |
| PNL-02/4 | 15.80 | 9.30 | 59.60 | 92.70 | 57 | 47.2 | 12.0 |
| PNL-02/5 | 21.80 | 11.58 | 83.50 | 96.80 | 64 | 41.3 | 13.2 |
| PNL-02/6 | 20.70 | 11.42 | 80.80 | 104.20 | 65 | 40.7 | 13.5 |
| PNL-02/7 | 18.20 | 10.65 | 74.40 | 115.40 | 48 | 49.4 | 16.5 |
| PNL-02/8 | 17.90 | 10.88 | 78.80 | 93.10 | 48 | 46.6 | 19.3 |
| PNL-03/1 | 20.00 | 11.72 | 79.10 | 121.90 | 50 | 43.1 | 12.0 |
| PNL-03/2 | 15.10 | 9.26 | 59.90 | 104.60 | 48 | 44.7 | 12.6 |
| PNL-03/3 | 14.50 | 8.55 | 54.80 | 88.80 | 51 | 47.8 | 11.8 |
| PNL-03/4 | 14.80 | 8.93 | 56.60 | 85.90 | 55 | 46.2 | 11.0 |
| PNL-03/5 | 15.00 | 8.96 | 55.60 | 87.90 | 54 | 49.2 | 10.4 |
| PNL-03/6 | 14.80 | 9.15 | 60.10 | 93.40 | 56 | 48.3 | 10.4 |
| PNL-03/7 | 15.70 | 9.11 | 59.00 | 91.70 | 54 | 48.0 | 12.2 |
| PNL-03/8 | 18.30 | 10.14 | 70.50 | 117.20 | 45 | 29.6 | 35.5 |
| RBL-01/1 | 15.90 | 10.37 | 75.10 | 101.60 | 47 | 21.0 | 26.8 |
| RBL-01/2 | 14.40 | 7.74 | 58.90 | 91.90 | 52 | 5.3 | 32.6 |
| RBL-01/3 | 13.90 | 8.55 | 58.20 | 85.70 | 50 | 12.0 | 33.0 |
| RBL-01/4 | 13.80 | 8.62 | 58.50 | 115.20 | 32 | 61.0 | 12.0 |
| RBL-01/5 | 13.90 | 8.49 | 57.70 | 99.20 | 42 | 43.8 | 17.5 |
| RBL-02/1 | 14.10 | 7.83 | 48.40 | 81.90 | 51 | 20.3 | 31.7 |
| RBL-02/2 | 13.90 | 8.12 | 56.20 | 89.60 | 45 | 7.5 | 37.1 |
| RBL-02/3 | 13.90 | 8.41 | 54.80 | 99.40 | 40 | 45.7 | 20.0 |
| RBL-03/1 | 11.43 | 7.05 | 42.00 | 73.00 | 44 | 15.2 | 35.9 |
| RBL-03/2 | 13.60 | 7.48 | 54.10 | 83.40 | 46 | 4.8 | 39.5 |
| RBL-03/3 | 10.65 | 7.12 | 43.80 | 98.10 | 33 | 51.7 | 18.4 |
| RBL-03/4 | 12.24 | 7.48 | 45.70 | 99.80 | 35 | 50.3 | 17.4 |

| Soil sample | EC1:5 dS $m^{-1}$ | EC1:10 dS $m^{-1}$ | Cl1:10 meq $L^{-1}$ | ECe dS $m^{-1}$ | SP % | Gypsum % | CCE % |
|---|---|---|---|---|---|---|---|
| RLL-01/1 | 21.90 | 12.55 | 95.40 | 119.50 | 51 | 41.2 | 19.8 |
| RLL-01/2 | 17.80 | 10.64 | 76.00 | 119.30 | 48 | 41.1 | 21.7 |
| RLL-01/3 | 16.80 | 9.70 | 66.30 | 101.20 | 48 | 43.9 | 19.7 |
| RLL-01/4 | 12.12 | 7.83 | 48.50 | 93.50 | 41 | 51.3 | 18.6 |
| RLL-01/5 | 13.90 | 8.46 | 54.20 | 108.10 | 32 | 55.7 | 15.3 |
| RLL-03/1 | 16.60 | 9.72 | 64.40 | 126.90 | 37 | 65.9 | 10.1 |
| RLL-03/2 | 14.40 | 9.39 | 62.60 | 114.90 | 35 | 64.3 | 11.7 |
| RLL-03/3 | 13.40 | 8.21 | 50.60 | 103.10 | 36 | 65.7 | 13.7 |
| RLL-03/4 | 14.00 | 8.40 | 53.20 | 111.00 | 34 | 64.9 | 10.9 |
| RLL-03/5 | 12.74 | 7.90 | 50.20 | 120.20 | 28 | 69.2 | 7.5 |
| RLL-04/1 | 9.21 | 6.17 | 33.10 | 70.60 | 39 | 60.5 | 13.7 |
| RLL-04/2 | 10.47 | 7.08 | 43.00 | 87.10 | 37 | 60.4 | 13.1 |
| RLL-04/3 | 10.05 | 6.29 | 34.30 | 95.70 | 34 | 66.5 | 13.4 |
| RLL-04/4 | 10.79 | 6.10 | 32.70 | 85.40 | 36 | 60.9 | 16.3 |
| RLL-04/5 | 8.97 | 6.90 | 39.70 | 80.80 | 31 | 61.7 | 16.5 |
| RLL-04/6 | 10.77 | 6.76 | 40.10 | 103.50 | 28 | 57.8 | 18.5 |
| RLL-05/1 | 12.72 | 7.96 | 49.40 | 104.50 | 31 | 45.6 | 19.4 |
| RLL-05/2 | 12.04 | 7.48 | 45.70 | 93.40 | 35 | 57.7 | 14.2 |
| RLL-05/3 | 12.42 | 7.49 | 46.10 | 105.20 | 32 | 71.9 | 9.4 |
| RLL-05/4 | 9.85 | 6.09 | 34.80 | 92.30 | 29 | 69.9 | 15.0 |
| RLL-05/5 | 11.39 | 7.44 | 46.20 | 103.90 | 30 | 43.1 | 30.0 |
| RLL-05/6 | 9.86 | 6.15 | 34.00 | 101.70 | 29 | 25.6 | 52.1 |
| RLL-06/1 | 10.74 | 6.96 | 40.30 | 87.90 | 35 | 35.4 | 27.8 |
| RLL-06/2 | 8.75 | 5.91 | 29.50 | 71.00 | 37 | 54.6 | 17.5 |
| RLL-06/3 | 7.55 | 5.27 | 23.70 | 66.10 | 30 | 41.1 | 30.5 |
| RLL-07/2 | 2.75 | 2.52 | 0.40 | 8.29 | 31 | 63.7 | 14.9 |
| RLL-07/3 | 3.07 | 2.64 | 1.50 | 14.50 | 27 | 65.1 | 16.6 |
| RLL-07/4 | 3.86 | 3.12 | 6.40 | 22.70 | 30 | 38.9 | 45.9 |
| RLL-07/5 | 3.70 | 2.99 | 5.40 | 24.40 | 28 | 21.3 | 69.3 |
| RLL-08/1 | 2.30 | 2.24 | 2.00 | 2.94 | 35 | 32.9 | 31.9 |
| RLL-08/2 | 2.48 | 2.35 | 2.30 | 3.99 | 40 | 28.1 | 28.6 |
| RLL-08/3 | 3.06 | 2.65 | 4.90 | 9.14 | 42 | 13.6 | 36.5 |
| RLL-09/1 | 2.62 | 2.39 | 3.10 | 5.98 | 39 | 22.4 | 31.5 |
| RLL-09/2 | 3.53 | 2.93 | 7.00 | 14.50 | 40 | 20.1 | 40.1 |
| RLL-09/3 | 3.58 | 2.95 | 6.70 | 17.40 | 39 | | |
| RLL-10/1 | 2.99 | 2.65 | 3.40 | 10.50 | 35 | 63.9 | 17.3 |
| RLL-10/2 | 3.42 | 2.84 | 4.50 | 18.60 | 29 | 72.7 | 13.0 |
| RLL-10/3 | 3.70 | 2.99 | 5.90 | 20.10 | 30 | 44.4 | 35.0 |
| RLL-10/4 | 3.98 | 3.15 | 7.20 | 24.40 | 31 | 28.5 | 57.5 |
| RLL-10/5 | 4.03 | 3.15 | 8.20 | 27.20 | 28 | 32.7 | 56.9 |
| RLL-11/1 | 2.34 | 2.29 | 1.20 | 3.33 | 38 | 22.7 | 36.9 |
| RLL-11/2 | 2.66 | 2.39 | 3.60 | 6.68 | 40 | 7.3 | 48.5 |
| RLL-11/3 | 3.12 | 2.63 | 4.20 | 10.05 | 36 | 11.1 | 53.7 |
| RLL-11/4 | 2.82 | 2.49 | 2.50 | 8.37 | 33 | 47.6 | 36.2 |

| Soil sample | EC1:5<br>dS $m^{-1}$ | EC1:10<br>dS $m^{-1}$ | Cl1:10<br>meq $L^{-1}$ | ECe<br>dS $m^{-1}$ | SP<br>% | Gypsum<br>% | CCE<br>% |
|---|---|---|---|---|---|---|---|
| RLL-12/1 | 2.29 | 2.06 | 2.10 | 3.03 | 37 | 4.9 | 46.5 |
| RLL-12/2 | 2.61 | 2.40 | 2.30 | 5.51 | 39 | 33.4 | 38.6 |
| RLL-12/3 | 2.51 | 2.33 | 2.10 | 5.09 | 36 | 81.0 | 8.8 |
| RLL-12/4 | 2.55 | 2.34 | 2.60 | 5.92 | 32 | 72.0 | 16.3 |
| RLL-12/5 | 2.69 | 2.42 | 2.40 | 6.77 | 33 | 54.1 | 26.2 |
| RLL-13/1 | 2.33 | 2.20 | 2.20 | 2.84 | 42 | 4.5 | 48.9 |
| RLL-13/2 | 2.30 | 1.86 | 1.90 | 3.05 | 43 | 3.5 | 51.9 |
